# Meiotic crossovers revealed by differential visualization of homologous chromosomes using enhanced haplotype oligo‐painting in cucumber

**DOI:** 10.1111/pbi.14546

**Published:** 2024-12-11

**Authors:** Qinzheng Zhao, Zhenhui Xiong, Chunyan Cheng, Yuhui Wang, Xianbo Feng, Xiaqing Yu, Qunfeng Lou, Jinfeng Chen

**Affiliations:** ^1^ State Key Laboratory of Crop Genetics & Germplasm Enhancement and Utilization, College of Horticulture Nanjing Agricultural University Nanjing 210095 China

**Keywords:** meiotic crossovers, homologous chromosomes, oligo‐FISH, chromosome pairing behaviours, cucumber

## Abstract

The interaction dynamics of homologous chromosomes during meiosis, such as recognition, pairing, synapsis, recombination, and segregation are vital for species fertility and genetic diversity within populations. Meiotic crossover (CO), a prominent feature of meiosis, ensures the faithful segregation of homologous chromosomes and enriches genetic diversity within a population. Nevertheless, visually distinguishing homologous chromosomes and COs remains an intractable challenge in cytological studies, particularly in non‐model or plants with small genomes, limiting insights into meiotic dynamics. In the present study, we developed a robust and reliable enhanced haplotype oligo‐painting (EHOP) technique to image small amounts of oligos, enabling visual discrimination of homologous chromosomes. Using EHOP developed based on sequence polymorphisms and reconstructed oligonucleotides, we visually distinguished parental and most recombinant chromosomes in cucumber F_1_ hybrids and F_2_ populations. Results from EHOP revealed that meiotic CO events preferentially occur in the 30–60% intervals of chromosome arms with lower sequence polymorphisms and significant recombination bias exists between cultivated and ancestral chromosomes. Due to the occupation of extensive heterochromatin occupancy, it is not yet possible to precisely identify the meiotic COs present in the central portion of chr2 and chr4. Notably, CO accessibility was universally detected in the cytological centromere region in F_2_ populations, a feature rarely observed in crops with large genomes. EHOP demonstrated exceptional performance in distinguishing homologous chromosomes and holds significant potential for broad application in studying homologous chromosome interactions.

## Introduction

Meiosis is a conserved eukaryotic cell division where homologous chromosomes pair and synapse to form bivalents (Mercier *et al*., 2015). A critical aspect of meiosis is the meiotic crossover (CO), which ensures the faithful segregation of bivalents and generates genetic diversity within the population by creating homologous chromosomal exchanges, a key factor in both plant and animal breeding. Meiotic recombination is triggered by programmed DNA double‐strand breaks (DSBs) in paired homologous chromosomes (Keeney and Neale, 2006). However, only a small fraction of meiotic DSBs result in COs, whilst most are repaired to non‐COs (NCOs) (Zelkowski *et al*., 2019). The fraction of DSBs repaired as COs and the number of COs per bivalent show significant differences amongst species, reflecting diversity in meiotic mechanisms.

CO events are not evenly distributed along chromosomes instead, they form distinct hotspots (Fernandes *et al*., 2019; Zelkowski *et al*., 2019). In crops with large genomes, CO hotspots tend to cluster in subtelomeric regions, away from the centromeres (Dreissig *et al*., 2019; Lukaszewski and Curtis, 1993; Mézard, 2006; Rodgers‐Melnick *et al*., 2015; Rommel Fuentes *et al*., 2020). Centromeres and pericentromeric regions exhibit strong CO suppression, with extensive heterochromatin occupying over half of the chromosomes. Plant centromeres and proximity regions typically consist of large tandem repeats and retrotransposons, which are believed to inhibit meiotic recombination (Fernandes *et al*., 2019). However, the factors limiting CO formation in these regions remain poorly understood, and direct cytological observations of meiotic COs in plant centromeres are lacking due to limited appropriate experimental materials and visualization tools.

Various approaches have been used to study meiotic COs in crops and model plants, including cytological investigation, immunostaining, segregation assays of genetic markers, and next‐generation and long‐read sequencing. Each approach has strengths and weaknesses in measuring COs (Kim and Choi, 2022). Recombination nodules (RN) and CO‐associated proteins have been applied primarily in several model plants, but they are less effective in non‐model plants and often fail to identify individual chromosomes (Durand *et al*., 2022; Ziolkowski *et al*., 2017). Pollen‐ and F_2_ population‐based genotyping allows high‐throughput detection of fine‐scale COs genome‐wide mapping, yet is limited by the quality of the reference genome assembly (Blackwell *et al*., 2020; Choi *et al*., 2017; Rommel Fuentes *et al*., 2020; Salomé *et al*., 2012; Si *et al*., 2015). Cytological analysis is a powerful and indispensable tool for visually assessing chromosome behaviour and evolution (Yang *et al*., 2019). Fluorescence *in situ* hybridization based on oligonucleotides (Oligo‐FISH) has shown unique advantages in identifying individual chromosomes, resolving chromosome evolution, and visualizing chromosome pairing, but fails to differentiate the homologous chromosomes (Jiang, 2019; Li *et al*., 2021; Zhao *et al*., 2021). Visual distinction of homologous chromosomes using oligo‐FISH was first achieved by querying single nucleotide polymorphisms (SNPs) in maize (do Vale Martins *et al*., 2019). However, this technique still requires dense oligo coverage limiting its applicability in species with low polymorphisms or small genomes, where few sequence differences are available to develop haplotype oligos.

The cucumber (*Cucumis sativus*, 2*n* = 2*x* = 14) is an economically important vegetable crop with four cross‐compatible botanical varieties, including the wild cucumber (*C. sativus* var. hardwickii), the semi‐wild Xishuangbanna cucumber (*C. sativus* var. *xishuangbannesis*), the Sikkim cucumber (*C. sativus* var. *sikkimensis*), and the cultivated cucumber (*C. sativus* var. *sativus*) (Li *et al*., 2022). The cucumber genome is ~350 Mb, of which nearly 30% is composed of heterochromatic satellite and rDNA sequences substantially higher than in crops such as rice and watermelon (<5%) (Guan *et al*., 2024). Cucumber, as a representative of small‐genome crops, has a narrow genetic background of its own and scarce genetic variation available for exploitation. However, there has been very restricted investigation of recombination characteristics in cucumber.

To visually distinguish homologous chromosomes and explore CO events in cucumber, we identified four sets of haplotype oligos pools by querying sequence divergence across two cultivated (*C. sativus* var. *sativus* cv. 9930 and cv. Gy14) and one ancestral cucumber (*C. sativus* var. *hardwickii*) genomes. Only a small number of differential sequences were available to develop haplotype oligo. The small amounts of oligo are insufficient to generate ideal FISH signals for distinguishing homologous chromosomes or localizing CO events. To overcome this, each oligo was designed with genomic sequence, flanking 35 bp non‐genomic signal enhancers (SEs), and PCR primers. The SEs recruited fluorophore‐labelled complementary SEs to achieve cascading enhancement of the oligo signals, enabling robust chromosome visualization. We successfully developed the enhanced haplotype oligo‐painting (EHOP) technique to achieve differential painting of homologous chromosomes with small amounts of haplotype oligos. This technique allowed us to characterize meiotic CO events and visualize meiotic homologous chromosome pairing in cucumber. Given its ability to work with limited sequence polymorphisms, EHOP holds significant potential for broader applications in other plants with small genomes.

## Results

### Development of enhanced haplotype oligo‐painting by recruiting signal enhancers

To develop haplotype‐specific oligo probes for recognizing homologous chromosomes from the parental lineage, we used the published strategy to generate single‐copy oligos (42 nucleotides (nt)) from the 9930 and Gy14 genome sequences (do Vale Martins *et al*., 2019). Those oligos with sequence diversity of ≥3 nt between Gy14 and 9930 genomes retained an average of 1000–3000 oligos per chromosome (0.03–0.1 oligo/kb) (Table S1). Due to the limited sequence variation in cucumber's small genome, this number of oligos was insufficient to generate optimal chromosome painting signals, where typically a higher number of oligos (density ≥0.1 oligo/kb) are required for decent oligo‐painting quality (Jiang, 2019).

**Figure 1:**
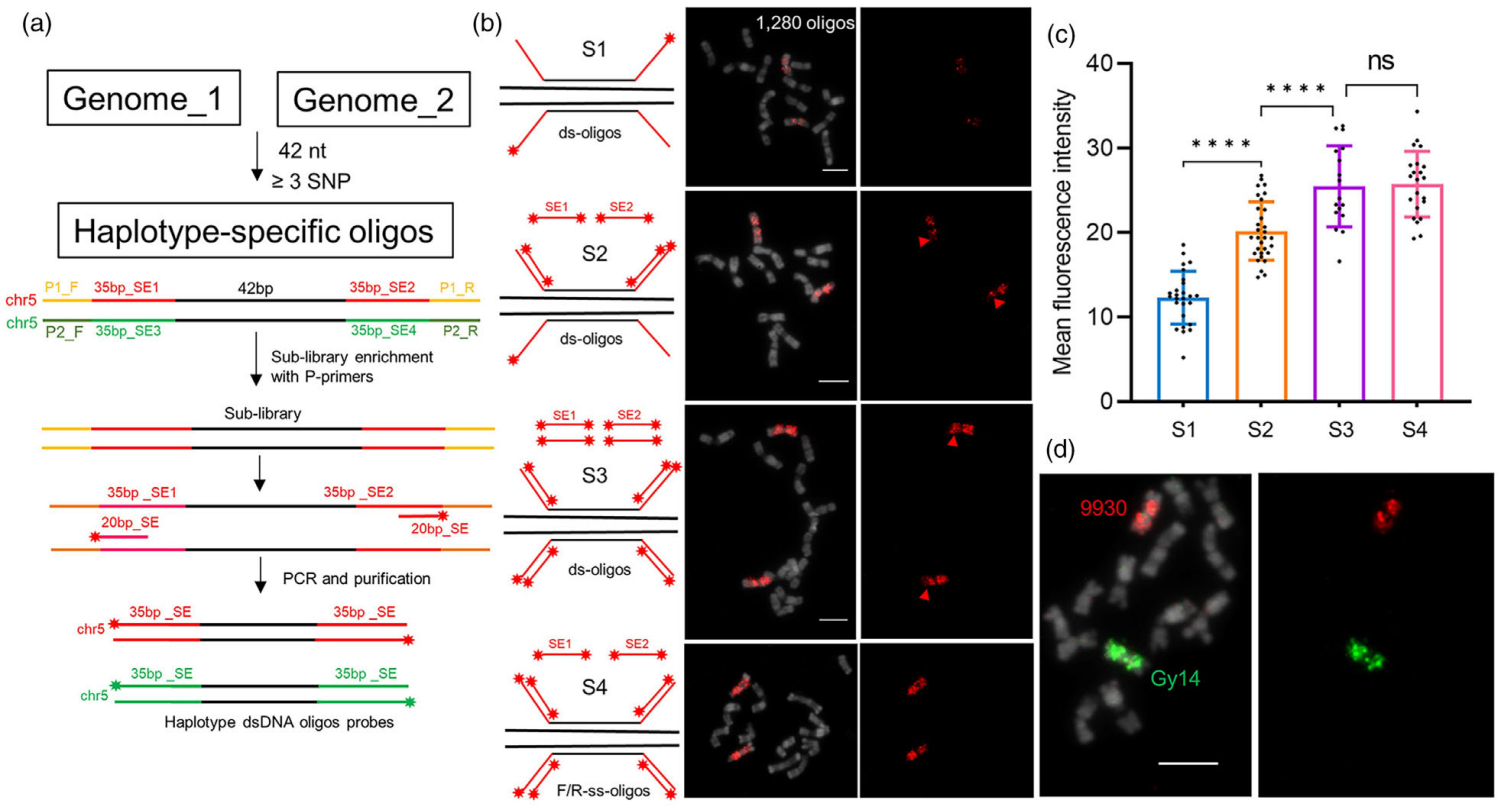
Development of enhanced haplotype oligo‐painting by recruiting signal enhancers. (a) Development strategy for enhanced haplotype oligo‐painting through oligo‐library reconstruction. Haplotype‐specific oligos flanked by 35 bp non‐genomic signal‐enhancers (SEs) sequences and PCR amplification primers (P_R/F). Each chromosome‐specific sub‐library was amplified from the total oligos library by using different P_R/F primers for PCR. Double‐stranded oligo probes were obtained by amplification using fluorescently labelled 20 bp SE primers based on the sub‐library as a template. (b) Four oligo‐painting strategies and FISH images based on the 9930‐chr5 probes. Strategy 1: standard oligo‐painting with double‐stranded oligo (ds‐oligo) probes. Strategy 2: enhanced oligo‐painting based on the ds‐oligos probes by recruiting two SEs. Strategy 3: enhanced oligo‐painting based on the ds‐oligos probes by recruiting two SEs and two complementary SEs. Strategy 4: enhanced oligo‐painting based on the forward and reverse‐strand oligos (F/R‐ss‐oligos) probes by recruiting two SEs. The red arrows indicate signals on the chr5 long arms. Enhanced haplotype oligo‐painting based on Strategy 3 named EHOP. (c) Comparison of mean fluorescence intensity among four different oligo‐painting strategies. Error bars indicate the ±SD over three biological replicates. Asterisks indicate statistically significant differences at *, ****P < 0.0001. (d) Differential painting of homologous chromosomes chr5 by EHOP in Gy14‐9930F_1_ hybrid. Bars = 5 μm.

To design an enhanced oligo‐library, we first focused on 9930‐chr5, which contained only 1280 oligos, the lowest density across chromosomes. Two different 35‐bp non‐genomic signal‐enhancers (SE1/2) sequences were attached to each oligo's forward‐ and reverse‐strand flanks, respectively (Figure 1a; Figure S1). When the oligo probes are hybridized to their target, the non‐genomic region will remain single‐stranded to recruit fluorescent‐labelled SE resulting in enhanced FISH signals. The designed oligos were further flanked adding 20 bp P‐primers to distinguish sub‐libraries by PCR amplification (Figure S1). We obtained double‐stranded oligo (ds‐oligo) probes and forward/reverse single‐stranded oligo (F/R‐ss‐oligos) probes of 9930‐chr5, respectively (see ‘Methods’ section). To assess the effectiveness of the SEs, we performed painting experiments on the mitotic chromosomes of 9930 with applying four strategies (Figure 1b). As a result, the 1280 ds‐oligo probes only failed to produce the desired painting signals (Figure 1b, Strategy 1). Two and four double‐ended TAMRA‐labelled 33 bp SEs were added during FISH experiments (Figure 1b, Strategy 2 and 3). Strategy 3 exhibits more desirable painting signals compared with Strategy 2, particularly in the long arm of chr5 showing more details even with only 443 oligos (Figure 1b, red arrows). Meanwhile, no significant painting differences were observed between the ds‐oligo and ss‐oligo probes applied in Strategy 3 and Strategy 4, respectively (Figure 1b, Strategy 3 and 4). Fluorescence intensity measurements confirmed that increased SE numbers significantly improved FISH signal quality, regardless of whether ds‐oligo or F/R‐ss‐oligo probes were used (Figure 1c). It indicates that non‐genomic regions of ds‐oligo probes also maintain the single strand unaffected by complementary strands (Figure 1c).

We tested the potential of Strategy 3 by mapping small DNA fragments or low‐density oligos. Four DNA regions (4, 8, 12, and 25 kb) from 9930‐chr7 were selected to generate 80, 150, 250, and 520 oligos, respectively (Figure S2). These regions all produced detectable FISH signals on mitotic chromosomes, with signal intensity becoming brighter as the number of oligos increased. Furthermore, the haplotype‐specific Gy14‐chr5 ds‐oligo probes were generated using FAM‐labelled SE3/4 primers. Two haplotype probes (9930‐chr5 and Gy14‐chr5) were hybridized to the metaphase chromosomes of Gy14‐9930F_1_ using Strategy 3. The result shows excellent haplotype signals, clearly and visually distinguishing maternal and paternal homologous chromosomes (Figure 1d). Our findings demonstrate that enhanced haplotype oligo‐painting based on Strategy 3, named EHOP, represents an accessible technique that works with ds‐oligo probes recruiting multiple SEs to increase the number of fluorophores on genomic targets, allowing the imaging of small amounts of oligos and visually distinguish homologous chromosomes with rare variants.

### Differential painting of homologous chromosomes using EHOP

Four haplotype‐specific oligo pools (hap9930‐hapGy14 and hap9930‐hapCuc64) containing 16 506, 16 111, 31 395, and 40 518 oligos, respectively were obtained using the methods described above to differentially paint homologous chromosomes from different cucumber varieties (Table S1). Four SEs and P‐primers were added to the obtained haplotype oligos flanks, respectively (Table S1). Using TAMRA (red) and FAM (green) labelled SE primers, a total of 28 chromosome‐specific haplotype ds‐oligo probes were harvested, 14 from hap9930‐hapGy14 and 14 from hap9930‐hapCuc64.

**Figure 2:**
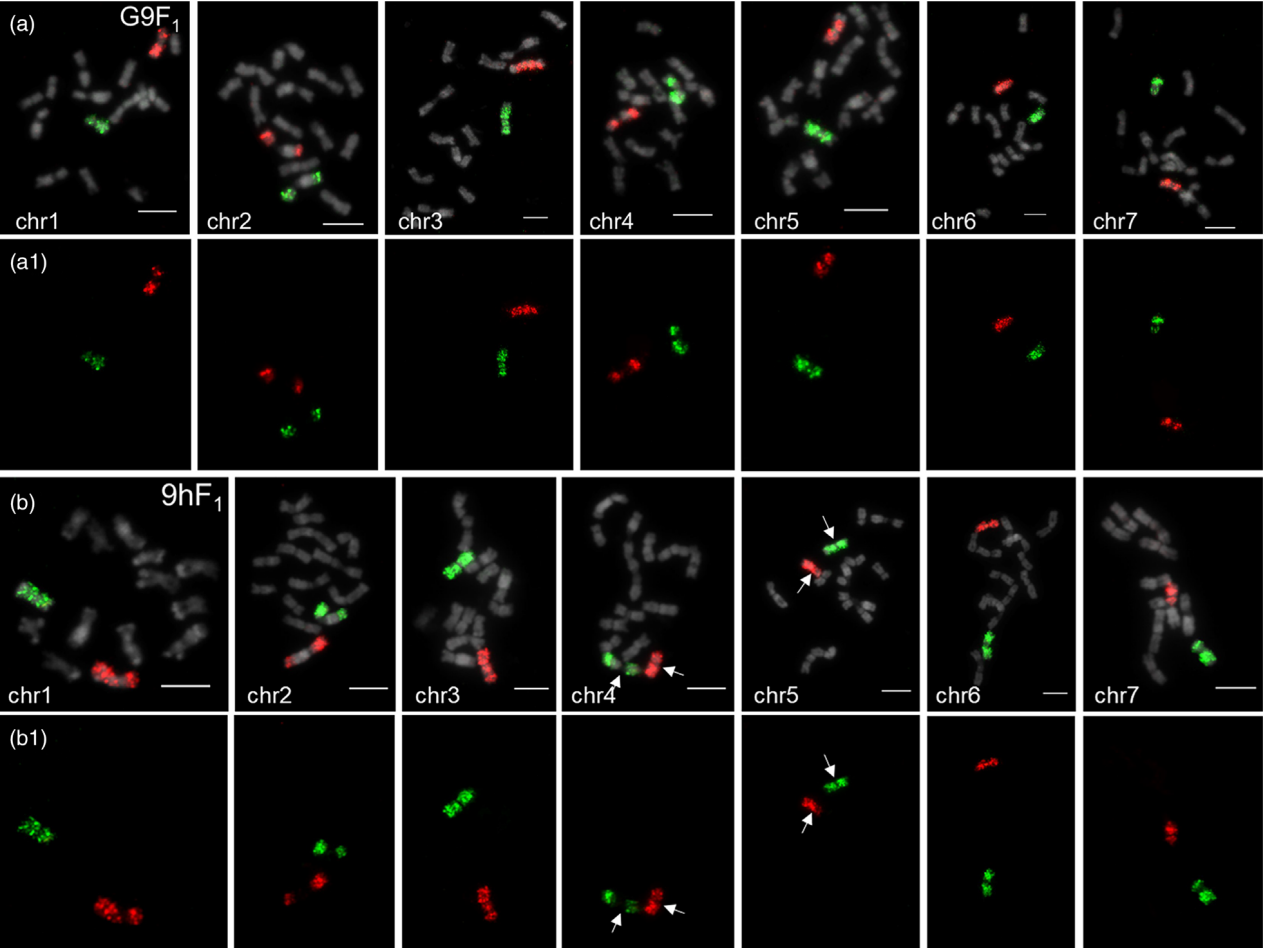
Differential painting of homologous chromosomes using EHOP in two cucumber hybrids. (a) Differential painting of seven homologous chromosomes by enhanced haplotype oligo‐painting (EHOP) on metaphase chromosomes of Gy14‐9930F_1_ hybrids (G9F_1_), respectively. Oligo‐painting probes specific to the 9930 haplotype were detected in red colour. Oligo‐painting probes specific to the Gy14 haplotype were detected in green colour. (a1) Merged painting signals derived from both 9930 and Gy14. (b) Differential painting of seven homologous chromosomes by EHOP on metaphase chromosomes of 9930‐hardwickii F_1_ hybrids (9hF_1_), respectively. Oligo‐painting probes specific to the hardwickii haplotype were detected in red colour. Oligo‐painting probes specific to the 9930 haplotype were detected in green colour. (b1) Merged painting signals derived from both hardwickii and 9930. White arrows indicate karyotypic differences in chr4 and chr5 due to domestication inversions. Bars = 5 μm.

Corresponding probe pools and SE from the identical chromosomes were hybridized to the metaphase chromosomes prepared from Gy14‐9930 and 9930‐hardwickii F_1_ hybrids (designated as G9F_1_ and 9hF_1_) by EHOP, respectively (Figure 2). Each oligo pool generated highly desirable haplotype painting signals that could visually distinguish maternal or paternal homologous chromosomes (Figure 2). However, no painting signals were detected in the central portion of chr2 and chr4 owing to extensive heterochromatin occupancy resulting in the absence of oligo probes (Figure 2, chr2 and chr4). Very weak cross‐hybridization signals were detected only on several chromosomes, despite only 3 nt differences between homologous oligos (Figures S3 and S4). The average density of hap9930‐hapCuc64 probes was 0.11–0.23 oligo/kb and produced stronger signals than those for hap9930‐hapGy14 (0.03–0.1 oligo/kb) (Table S1). Owing to the karyotypic differences caused by the inversion between 9930 and hardwickii (Zhao *et al*., 2021), these are significant haplotype painting differences on chromosomes chr4 and chr5 (Figure 2b, white arrows). These results suggest that EHOP can be an effective tool for exploring homologous chromosome recombination and pairing in cucumber.

### Meiotic COs characteristics of homologous chromosomes of cultivated cucumber

**Figure 3:**
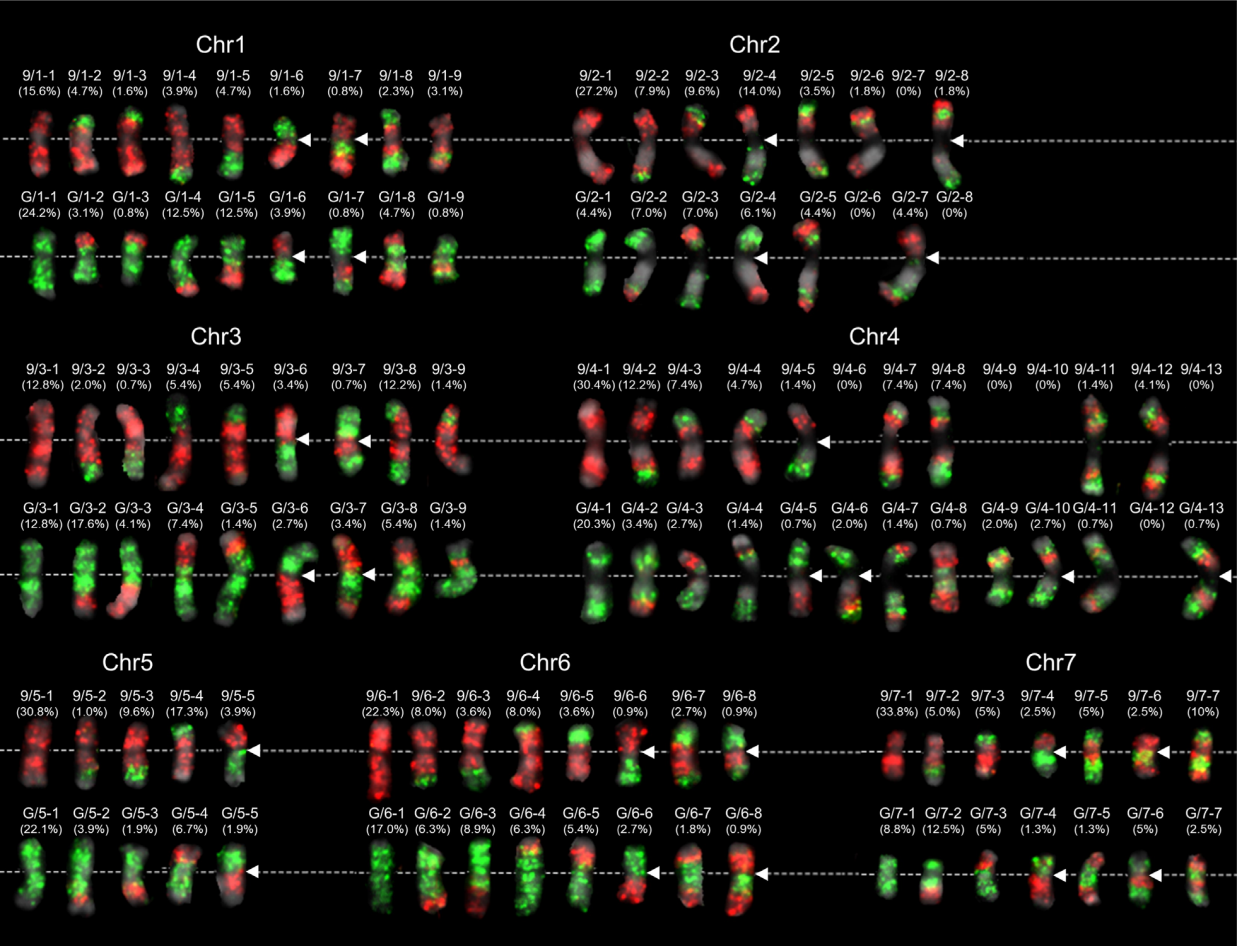
Meiotic CO patterns and frequencies of homologous chromosomes revealed by EHOP in the Gy14‐9930F_2_ population. Each enumerated meiotic CO pattern was taken from Figure S5 and arranged in increasing order of the CO number. The frequency of each pattern was calculated by dividing the number of that pattern by the total number of chromosome copies analysed. The data are presented in the upper brackets of recombination chromosomes. The complementary recombination patterns are divided into two rows, with chromosome 9930 in the top row and Gy14 in the bottom. White dashed lines mark the positions of the cytological centromeres. White arrows indicate the observed CO events at cytological centromere (primary constriction) regions.

Given the excellent ability of EHOP in differentially painting chromosomes at low oligo densities, we further investigated the meiotic COs distribution and recombination patterns of cucumber chromosomes. We performed large‐scale EHOP experiments on somatic metaphase chromosomes from Gy14‐9930F_2_ plants using the seven hap9930 and seven hapGy14 probe pools. Each F_2_ plant contained two copies of each chromosome, each classified as parental or recombinant based on the FISH pattern (Figure S3). We enumerated the meiotic CO patterns of all seven cucumber chromosomes and the frequency of each pattern based on the positions of chromosomal exchanges (Figure 3). These results showed recombination patterns ranging from 5 to 13 per chromosome (Figure 3).

However, certain patterns were absent including two recombination patterns of chr2 and four recombination patterns of chr4 (9/4–9, 9/4–10, 9/4–13, and G/4–12). This phenomenon may be due to the limited number of populations analysed or the genetic drag of certain recombination combinations that may negatively affect gamete developmental or progeny viability. We calculated the frequency of chromosomal exchanges per chromosome generated by meiotic COs, ranging from 47.1% to 74.4%, with chr5 having the lowest recombination frequency and chr3 the highest at 74.4%. These variations indicate the different potential of each chromosome to generate genetic variation through COs. The single CO events were the most common, accounting for over 54.5% of the recombined chromosomes analysed. Double CO events in the same arm were found in ~2.8% to 12.5% of the analysed chromosomes, except for chr5 and chr6. Three CO events were observed only on chr2, chr4, and chr7, representing 1.8%, 6.9%, and 12.5% frequency. Particularly, the four CO events were identified in 148 copies of chr4 analysed. Notably, widespread meiotic CO events were identified in seven chromosomes' primary constriction regions (cytological centromeres) (Figure 3; Figure S5, white arrows).

**Figure 4:**
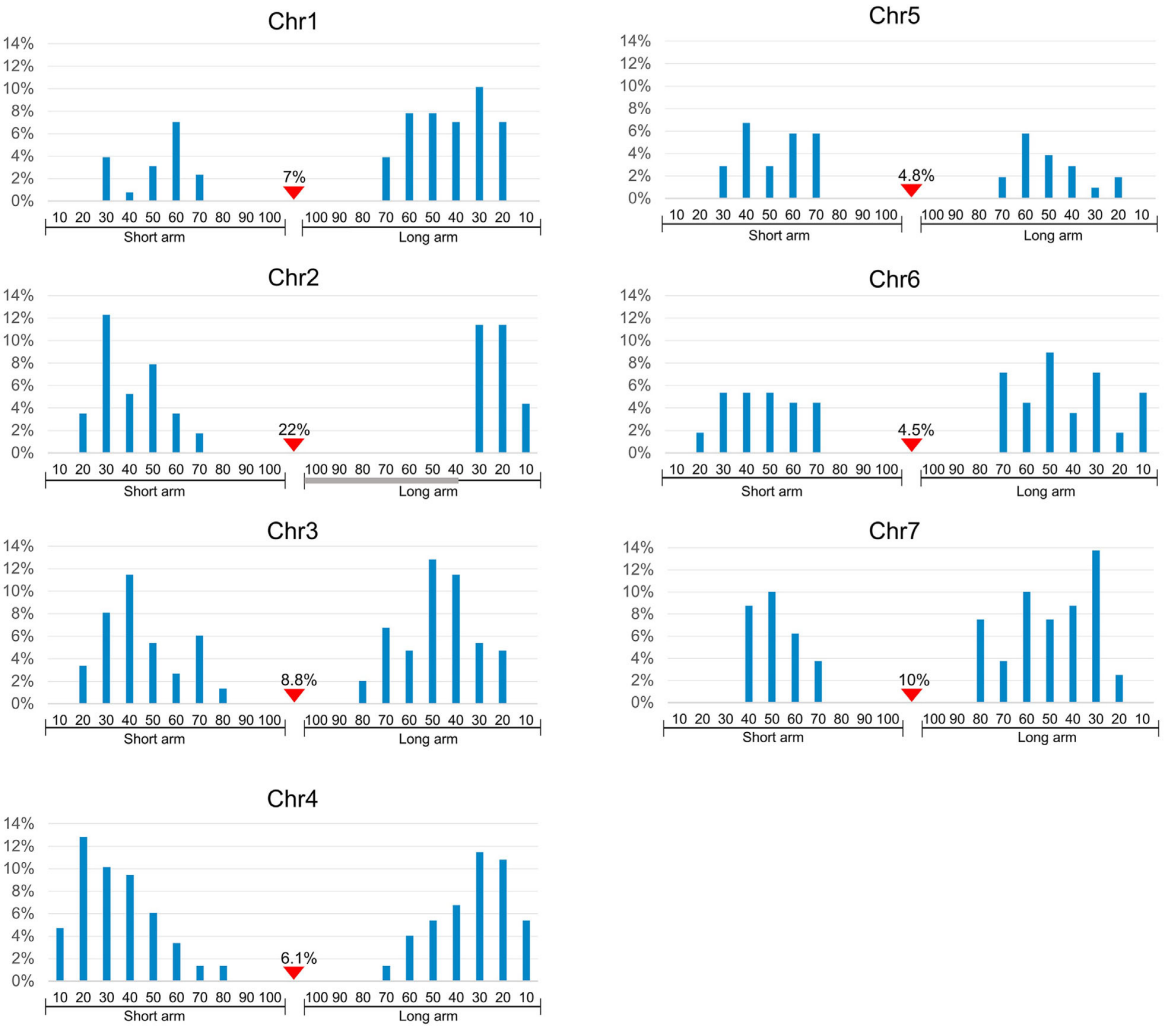
Distribution and frequencies of meiotic COs on Gy14‐9930 chromosomes. Each chromosome arm was divided into 0–100 intervals. Each meiotic CO from Figure S5 was measured and positioned to the corresponding interval. Each interval on the *x*‐axis represents 10% of the length of the short or long arm. The *y*‐axis indicates the frequencies of COs within a particular 10% of the short or long arm. The CO frequencies were calculated by dividing the CO in each interval by the number of chromosomes analysed. The bold grey line indicates the heterochromatin region located in the long arm of chr2. The red triangles point to the cytological centromere positions on the chromosomes and COs frequencies.

We characterized the distribution of each CO on the chromosomes as previously described (do Vale Martins *et al*., 2019). The short and long arms of seven chromosomes were evenly divided into 100 intervals, from 0 at the telomere to 100 at the centromere (Figure 4). The frequency of mapped COs occurring in each interval was calculated (Figure 4). Most COs were located within the 20–70 intervals on both the short or long arms. High‐frequency CO events were detected in the 30–60 intervals on both the short and long arms, accompanied by a decreasing trend towards centromere. The long arm holds more CO events than the short arm, especially for chr1, chr6, and chr7, which have higher arm ratios (Figure 4; Table S2). Only ~27.2% COs were observed within the 10–30 intervals of the chr2 long arm, lower than that on the short arm with a number of the 34.2%. The 70% intervals near the centromere of the chr2 long arm are covered by heterochromatin, resulting in a shorter effective chromosome axis (Figure 4 chr2). Centromere‐proximal COs were also frequent, with occurrences ranging from 4.5% to 10% on most chromosomes, but up to 22% on chr2 (Figure 4, red arrows). Overall, meiotic COs of cucumber homologous chromosomes were characterized cytologically using the EHOP, and the results showed that its CO characteristics were marked by cytological centromeres with the accessibility of meiotic COs.

### Meiotic COs characteristics between cultivated and ancestral chromosomes

**Figure 5:**
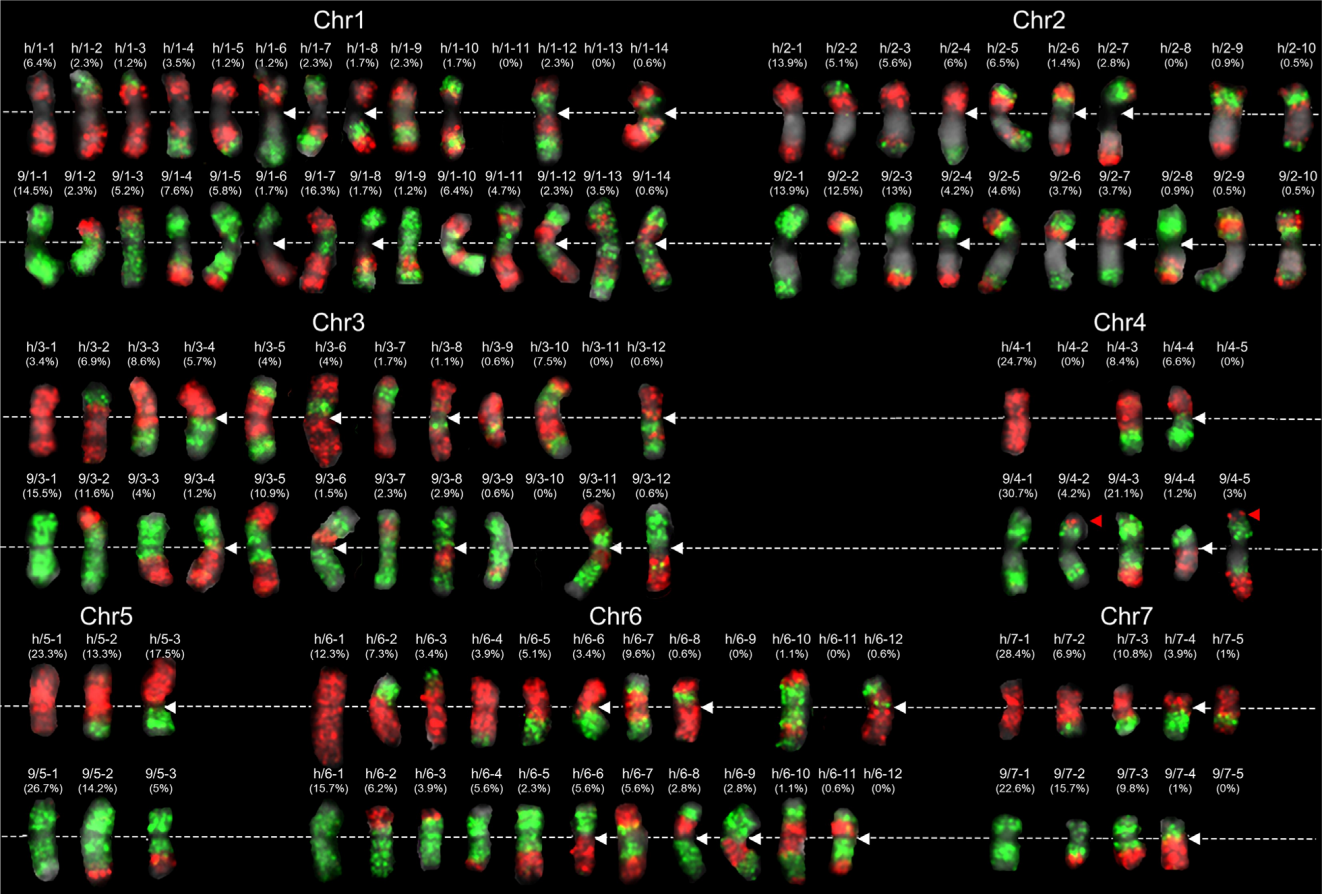
Meiotic CO patterns and frequencies of ancestral and cultivated chromosomes revealed by EHOP in hardwickii‐9930F_2_ population. Each enumerated meiotic CO pattern was taken from Figure S7 and arranged in increasing order of the number of COs. The frequency of each pattern was calculated by dividing the number of that pattern by the total number of chromosome copies analysed. The data are presented in the upper brackets of recombination chromosomes. The complementary recombination patterns are divided into two rows, with chromosome hardwickii in the top row and 9930 in the bottom. White dashed lines mark the positions of the cytological centromeres. White arrows indicate that the observed chromosome exchange positions are in cytological centromere (primary constriction) regions. Red arrows indicate the haplotype sequence signals of hardwickii that were detected in the telomere‐proximal region of the short arm of 9930‐chr4.

We further explored the recombination differences between cultivated cucumber (cv. 9930) and its ancestral wild relative, *C. sativus* var. hardwickii that represents a large amount of undomesticated genetic variation. These genetic variants can be introduced into cultivated cucumbers by meiotic recombination. Previously, we have inferred the evolutionary events of cultivated cucumber and wild hardwickii karyotypes by reconstructing the ancestral karyotypes (Zhao *et al*., 2021). Hardwickii underwent two inversions on the chr4 long arm, whereas cultivated cucumber underwent four inversions on the chr4, chr5, and chr7 (Figure S6). However, the recombination characteristics between ancestral and cultivated chromosomes have not been investigated. Therefore, we performed large‐scale EHOP experiments on somatic metaphase chromosomes from more than 200 F2 plants, which were derived from selfing 9930‐hardwickii F_1_ (9hF_1_) and hardwickii‐9930F_1_ (h9F_1_) hybrids using the seven hap9930 (green) and seven hapCuc64 (red) probe pools. As described above, we also equally enumerated the meiotic CO patterns of ancestral and cultivated chromosomes and the frequency of each pattern (Figure 5; Figure S7).

The results showed that chr1, chr2, chr3, and chr6 chromosomes exhibited 10–14 distinct recombination patterns. In contrast, chr4, chr5, and chr7, which contain inversion regions, displayed significantly few patterns, with only three to five recombination patterns (Figure S7), significantly fewer than those detected in G9F_2_. We note that each chromosome except chr5, lacked one or two recombination patterns (Figure 5), which commonly involved chromosome exchange or two meiotic COs on the same arm. These missing patterns indicate that such recombinations may be rare or possibly detrimental to gamete developmental or progeny viability. The recombination frequencies were calculated for each chromosome, ranging from 44.6% to 81.1%. Specifically, the recombination frequencies of chr4, chr5, and chr7 were 44.6%, 50%, and 49%, respectively, significantly lower than those observed in the other chromosomes (ranging from 72% to 81.1%). This suggests that large inversion events significantly inhibit the COs formation. Furthermore, we observed that cultivated chromosomes were preferentially inherited compared with ancestral chromosomes, with cultivated chromosomes chr1, chr2, chr3, and chr4 constituting 73.3%, 57.3%, 55.9%, and 60.3% of the analysed chromosomes, respectively (Figure 5). This phenomenon highlights a strong chromosomal inheritance bias in cucumbers. Additionally, eight of the 11 absent patterns involved ancestral chromosomes, further indicating the existence of this inheritance bias. To ensure this bias was not a result of hybridization direction, we examined the ratio of ancestral to cultivated chromosomes in 9hF_2_ and h9F_2_ populations, confirming that cultivated chromosomes are predominated in both 9hF_2_ and h9F_2_ (Figure S7).

**Figure 6:**
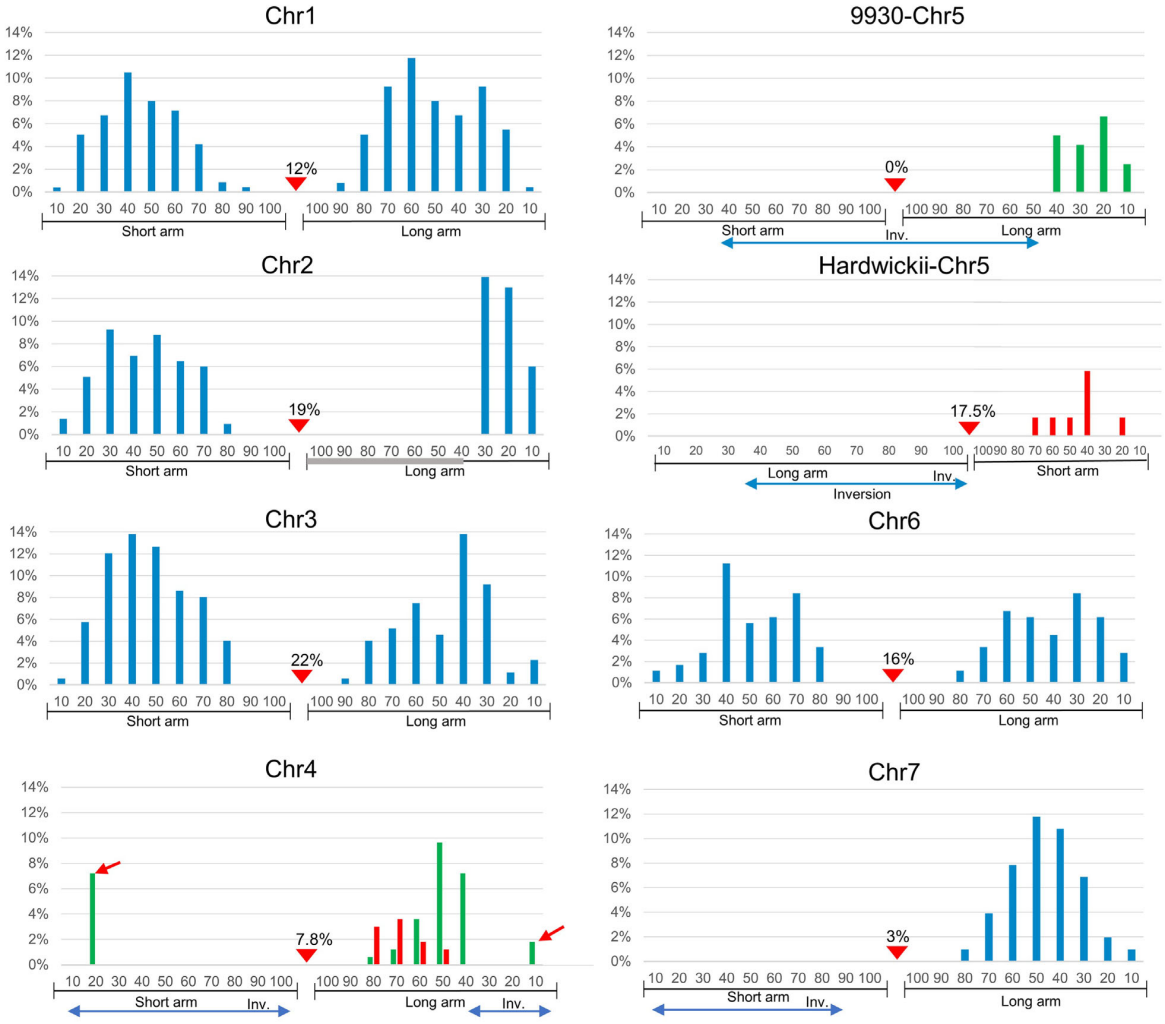
Distribution and frequencies of meiotic COs on ancestral and cultivated chromosomes. Each chromosome arm was divided into 0–100 intervals. Each meiotic CO from Figure S7 was measured and positioned to the corresponding interval. Each interval on the *x*‐axis represents 10% of the length of the short or long arm. The *y*‐axis indicates the frequencies of COs within a particular 10% of the short or long arm. The CO frequencies were calculated by dividing the CO in each interval by the number of chromosomes analysed. The bold grey line indicates the heterochromatin region located in the long arm of chr2. The blue double‐arrowed line indicates the inverted region on the chr4, chr5, and chr7. The red triangles point to the cytological centromere positions on the chromosomes and CO frequencies. Red arrows indicate recombination events and frequencies detected in the 9930‐chr4 inversion interval.

The meiotic COs were mapped across chromosomal intervals, as described above. The frequency of COs occurring in each interval was calculated (Figure 6). High‐frequency CO events were concentrated between intervals 30–60 on both the short and long arms except in the regions with inversions, with a decreasing trend towards the centromeres and telomeres. Due to the inversion‐induced karyotypic differences, we plotted the COs distribution for chr4 and chr5 separately (Figure 6), where red bars represent ancestral hardwickii chromosomes and green bars represent cultivated 9930 chromosomes. Unexpectedly, CO events were detected in interval 20 of the short arm and interval 10 of the long arm of chr4 both within inversion regions (Figures 5 and 6, red arrows). Around 7.2% of the 9930 chromosomes (9/4–2 and 9/4–5) underwent unbalanced chromosomal exchanges, as their corresponding hardwickii chromosomes (h/4–2 and h/4–5) were absent in the 166 analysed chr4 copies. In contrast, no chromosomal exchanges were observed in the inversion regions of chr5 and chr7 (Figure 6).

We also noted varying frequencies of exchange positions near the cytological centromere regions, ranging from 3% to 22% across chromosomes (Figure 5, white arrows and Figure 6, red arrows). Resequencing data from two F_2_ plants demonstrated the occurrence of chromosomal exchanges in both the pericentromeric regions, cytologically visible as exchange positions located in the primary constriction zones (Figure S8). No exchange position was detected within the centromere region of 9930‐chr5, likely due to the protective effect of the inversion surrounding the centromere (Figure 6 chr5). Overall, these results indicate that the CO hotspots in cucumber homologous chromosomes were conserved but suppressed by inversions.

### COs accessibility prefers the low‐polymorphism regions on chromosome arms

**Figure 7:**
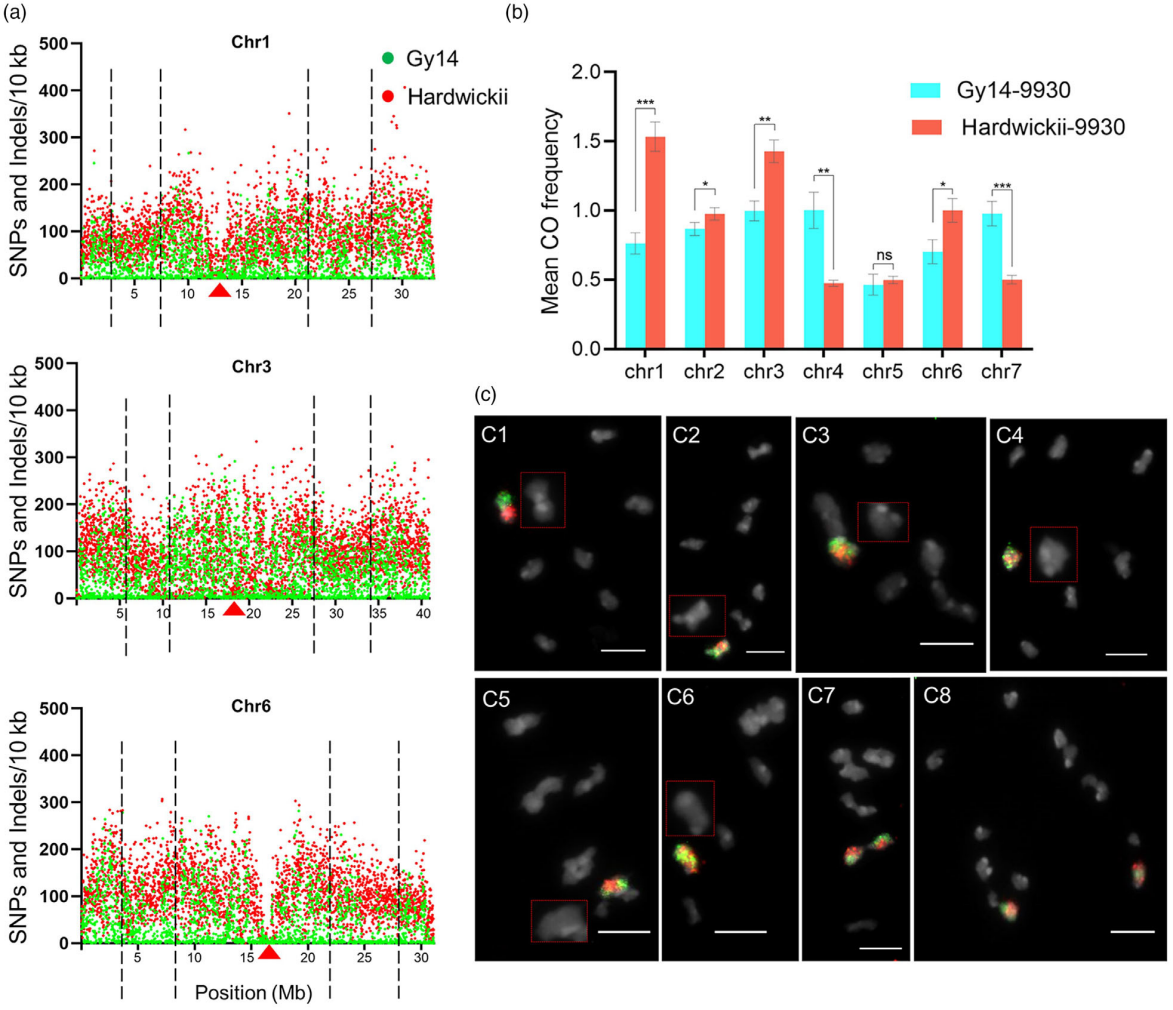
Sequence polymorphism characteristics and pairing behaviour visualization of cucumber homologous chromosomes. (a) Distribution characteristics of sequence polymorphisms (SNP and InDels) along chromosomes chr1, chr3, and chr6 of Gy14 and hardwickii. The black dashed lines indicate the chromosome arms' 30% or 60% intervals. The red triangles point to putative centromere regions. (b) Comparison of mean CO frequency per chromosome between two F_2_ populations (Gy14‐9930 and hardwickii‐9930). Error bars indicate the ±SD over three biological replicates. Asterisks denote significant differences between F_2_ populations according to T‐test with *P < 0.05; **P < 0.01; ***P < 0.001. (c) Visualization of chr1 meiotic pairing behaviour based on EHOP. (C1) A telomeres and telomeres rod‐bivalent with non‐chiasmatic. (C2) A rod‐bivalent with one chiasma on one arm only. (C3) A bivalent with two chiasmata on the same short or long arms. (C4) A ring‐bivalent with both arms bound by one chiasma. (C5) A complete telomere‐to‐telomere rod‐bivalent with three chiasmata. (C6) A complete telomere‐to‐telomere rod‐bivalent with four chiasmata. (C7) A recombinant bivalent undergoes initial segregation at the meiotic metaphase I. (C8) A recombinant divalent with post‐segregation at the meiotic anaphase I. Bars = 5 μm.

We were interested in how sequence variation impacts the accessibility of meiotic COs. Therefore, we analysed sequence variations in Gy14 and hardwickii using 9930v3 genome as a reference. A total of 666 161 and 1 773 108 sequence variations (SNPs and InDels) were identified in Gy14 and hardwickii as publicly available data (Li *et al*., 2022). These sequence variations were unevenly distributed along the chromosomes, with low sequence variation regions occupying approximately the 30%–60% interval of each arm (Figure 7a, between the black lines). These regions exhibited high frequencies of meiotic COs in the Gy14‐9930 and hardwickii‐9930F_2_ populations, whereas relatively low frequencies of meiotic COs were observed in regions with high sequence variations (Figures 4 and 6). These results suggest that meiotic COs preferentially occur in chromosome regions with lower sequence variation.

To further investigate the effect of sequence variation on COs in differentiated homologous chromosomes, we compared the mean CO frequency of each chromosome (Figure 7b). The results showed that the mean CO frequencies of chr1, chr2, chr3, and chr6 in the hardwickii‐9930F_2_ population were significantly higher than those in Gy14‐9930, whereas the mean CO frequencies of chr4 and chr7 were significantly suppressed by inversion events. The CO frequency of chr5 was the lowest in both F_2_ populations, with an average of only 0.5 CO events (Figure 7b). These results demonstrate that the sequence variations of divergent genomes contribute to increasing the overall COs accessibility of cucumbers in the absence of inversions. As noted above, the hardwickii‐9930 homologous chromosomes truly exhibited richer recombination patterns, broader COs distributions, and higher rates of multi‐COs events (Figure 3 to Figure 6).

### EHOP‐based visualization of pairing behaviours between homologues

During meiosis I, homologous chromosomes undergo dynamic interactions, including pairing, recombination, and segregation, which are key events ensuing that genetic information is shuffled between homologues, producing genetically diverse gametes. Meiotic chromosome behaviour has been extensively studied in both diploids and polyploids for their cytogenetic importance. Given the proven capability of EHOP in distinguishing mitotic homologues, we applied it to visualize the pairing behaviour of meiotic homologues using the chr1 probes of hap9930 and hapCuc64 (Figure 7c). Six bivalent categories were observed at metaphase I (Figure 7c1–c6), corresponding to the formation of zero to four chiasmata in chromosomes (Huang *et al*., 2022). In Category 1, homologues formed non‐chiasmatic telomeres and telomeres rod‐bivalent, confirmed by heterochromatic knobs and regular FISH signals (Figure 7c1). In Category 2, the exchanged FISH signals identified the rod‐bivalent, bound by one chiasma in one arm only (Figure 7c2). In Category 3, the same short or long arms of homologues form tightly paired structures that are thought to be bound by two chiasmata (Figure 7c3). In Category 4, ring‐bivalent were identified, with both arms bound by one chiasma (Figure 7c4). Categories 5 and 6 showed the complete telomere‐to‐telomere rod‐bivalent, bound by three and four chiasmata, respectively, as confirmed by the complexity of the FISH signals (Figure 7c5,c6). In addition, we observed the initial segregation and post‐segregation states of recombinant bivalents (Figure 7c7,c8). In conclusion, EHOP‐based meiotic analysis provides a visual tool for studying meiosis‐related mechanisms in diploids and polyploids.

## Discussion

There is considerable evidence to indicate that meiotic COs are not evenly distributed along chromosomes, instead forming different recombination hotspots in certain regions (Mézard, 2006). In large genome species, such as wheat, maize, barley, and tomato, recombination events typically concentrate in distal regions, away from centromeres (Dreissig *et al*., 2019; Lukaszewski and Curtis, 1993; Rodgers‐Melnick *et al*., 2015; Rommel Fuentes *et al*., 2020). Similarly, in cucumber, we also observed an uneven distribution of meiotic COs, with recombination rates increasing with distance from the centromeres and decreasing towards telomeres, akin to findings in rice (Si *et al*., 2015; Wu *et al*., 2003). In *Arabidopsis thaliana*, recombination frequencies vary between populations but consistently increase adjacent to the centromeres (Salomé *et al*., 2012). In cucumber, the 30–60% intervals of chromosome arms displayed high recombination frequencies, a pattern conserved across parental accessions (Figures 4 and 6). Notably, meiotic COs tended to occur in regions of low sequence divergence (Figure 7a). This variation in recombination frequency along chromosomes strongly correlates with sequence divergence, as polymorphism between homologous chromosomes can negatively affect meiotic COs formation (Lian *et al*., 2022).

A parabolic relationship between recombination frequency and SNP density has been observed in *Arabidopsis* hybrids. Recombination frequency and SNP density were positively correlated, but higher polymorphism density was associated with reduced recombination frequency (Blackwell *et al*., 2020). In our study, recombination frequencies and CO distribution were higher in hardwickii‐9930 than in Gy14‐9930, likely due to increased polymorphisms in hardwickii‐9930 (Figure 7b). This demonstrates that small‐scale or moderate sequence divergence densities promote meiotic recombination, whereas large‐scale (megabase‐scale) inversions completely suppress COs by preventing homologous unpairing in the inversion regions, as observed in the hardwickii‐9930F_2_ population (Figure 6). However, despite an inversion on the short arm of chr4 between 9930 and hardwickii, we detected low‐frequency, unbalanced, and small‐segment chromosomal exchanges at the ends of the 9930‐chr4 short arms (Figure 5; Figure S7). The absence of several recombination patterns, especially those carrying multiple COs that favoured ancestral chromosomes, could be due to insufficient F_2_ population sizes analysed or the detrimental nature of such recombination events.

The suppression of meiotic COs in centromere regions is a conserved phenomenon, as COs in these regions can cause chromosome missegregation and aneuploidy (Nambiar and Smith, 2016; Underwood *et al*., 2018). Surprisingly, we observed widespread chromosomal exchanges localized in the primary constriction regions of cucumber chromosomes by EHOP, with varying frequencies amongst different chromosomes (Figures 3, 4, 5, 6). Sequencing and genotyping of two F_2_ plants revealed that these exchange positions occurred near the genetically defined centromere regions (Figure S7). Cytologically, these exchange positions were observed in the primary constriction or pericentromeric regions, indicating that cucumber centromeres exhibit typical meiotic CO accessibility. This may be related to the evolutionary dynamics of the cucumber karyotype. In plants with large genomes, such as wheat, barley, tomato, and maize, centromeres are surrounded by transposon‐dense pericentromeric heterochromatin that occupies over half of the chromosome. In contrast, small‐genome plants such as *Arabidopsis* and rice have relatively low transposon content, mainly located around and within centromeric regions (Lambing *et al*., 2017). In *Arabidopsis*, disruption of the H3K9me2 and non‐CG DNA methylation pathways increases meiotic recombination in pericentromeric regions (Underwood *et al*., 2018). Likewise, manipulating alleles that drive CO formation can increase or release COs in recombination‐poor regions, enhancing genetic diversity for the advantageous in plant breeding (Blackwell *et al*., 2020; Mieulet *et al*., 2018; Taagen *et al*., 2020; Ziolkowski *et al*., 2017).

Conserved sequence‐based oligo‐FISH has been employed to study chromosome evolution and variation across plant species (Li *et al*., 2021; Zhao *et al*., 2021). Specific sequences from wild relatives have been used to develop oligo‐FISH probes for the identification of additional chromosomes and introgression sequences in chromosome engineering materials (Zhao *et al*., 2022). Traditional oligo‐FISH enhances the resolution of chromosomal structures and evolutionary studies, but it does not allow for visual distinction of maternal, paternal, or any homologous chromosomes (Beliveau *et al*., 2015). Different lineages often harbour rich sequence variations, implying the potential for a differential painting of homologous chromosomes owing to the high sensitivity of oligo probes. In mammalian and insect systems, maternal and paternal homologous chromosomes have been visually distinguished using haplotype oligo‐painting (HOP) developed by homologue‐based SNP (Beliveau *et al*., 2015). A haplotype‐specific oligo‐FISH based on maize chr10 was also developed and used for the visual painting of homologous chromosomes, the characterization of meiotic COs, and the analysis of meiotic pairing in maize (Braz *et al*., 2021; do Vale Martins *et al*., 2019).

For plants with large genomes, it is accessible to develop enough oligos to distinguish homologous chromosomes. However, in small‐genome plants like cucumber, the limited sequence variants pose a challenge for haplotype‐specific oligo development. To overcome this, we optimized the HOP method by adjusting oligos design parameters and reconstructing the oligos structure to enhance FISH signals through recruiting secondary oligos. We designated this optimized method as enhanced‐HOP (EHOP). With EHOP, FISH intensity increased significantly with the increasing number of secondary oligos, allowing the method to work effectively with a small amount of oligos in plants with small genome sizes. Using EHOP, we accurately targeted small regions (around 4 kb, containing ~80 oligos) on the chromosomes. We believe that the EHOP method shows potential for improving haplotype genome assembly accuracy and allows for differential painting of homologous chromosomes, facilitating detailed analysis of meiotic COs and pairing behaviour. Moreover, EHOP significantly reduces the density of oligo designs and the synthesis cost of the oligo‐library.

The RN‐ or CO protein‐based techniques have been used to predict and quantify the ‘future’ COs in synaptonemal complexes, they are limited to specific genotypes and have mainly been applied to model plants (Durand *et al*., 2022; Kim and Choi, 2022; Taagen *et al*., 2020; Wang *et al*., 2015). Additionally, genotyping methods based on the F_1_ hybrid pollen and F_2_ populations have been widely used to characterize meiotic recombination (Choi *et al*., 2017; Lian *et al*., 2022; Rommel Fuentes *et al*., 2020; Salomé *et al*., 2012; Wang *et al*., 2023). Haplotype oligo‐FISH complements these methods by offering insights into ‘past’ or historical COs resulting from sister chromatid exchange, though it may perform poorly in mapping COs at chromosome ends. Our proposed EHOP method addresses this limitation by enhancing FISH resolution. The use of haplotype oligo‐FISH to study meiotic pairing and recombination mechanisms is a significant advancement, as it can visually distinguish between parental chromosomes in the progeny and provide a direct view of meiotic homologues pairing. This is impossible to achieve with sequencing alone. By using minimal oligo probes, our technique could distinguish homologous chromosomes and locate very short genomic regions. This capability will significantly enhance our understanding of homologous recombination, chromosome pairing, intra‐ and inter‐chromosome interactions, and single‐allele spatial expression phenomena.

## Materials and methods

### Plant materials and chromosome preparation

Two cultivated cucumbers (*C. sativus* L. cv. 9930 and Gy14) and one ancestral cucumber (*C. sativus* var. *hardwickii*, Cuc64), which is the ancestor of the cultivated cucumber, were selected for the construction of F_1_ hybrids and F_2_ populations. About 9930 was chosen as a shared parent to cross with Gy14 and hardwickii, respectively. The harvested F_1_ plants were self‐pollinated to produce F_2_ offspring. F_1_ seedlings were planted in soil with standard field management under greenhouse conditions. The F_1_ and F_2_ seeds were treated in moist Petri dishes at 25 °C to obtain root tips. Root tips and young male anthers were treated and fixed in Carnoy's solution. The procedure of chromosome preparations was performed as described previously (Zhao *et al*., 2021).

### Development of haplotype secondary enhancement oligo‐painting library

Reference genomes of 9930v3, Gy14v2, and hardwickii (Cuc64) were downloaded from CuGenDBv2 and NCBI under project PRJNA657438, respectively (Li *et al*., 2022; Yu *et al*., 2023). Chorus software was used to generate single‐copy 42‐nt oligos for each genome with the parameters ‘‐l 42 ‐homology 80 ‐d 6 ‐step 3’ (Zhang *et al*., 2021). To obtain haplotype Gy14‐9930 oligos, 9930 (Gy14) oligos were aligned to the Gy14 (9930) reference genome using BWA ALN and BLAST software. Oligos with undefined and diversity ≥3 nt (Indel or SNP ≥3 nt) were extracted using python scripts, defining these oligos as 9930 and Gy14‐specific haplotype oligos. For 9930‐ hardwickii, the same process was used to obtain the corresponding haplotype oligos. Four oligo libraries (hap9930‐hapGy14 and hap9930‐hapCuc64) were obtained containing 16 506, 16 111, 31 395, and 40 518 oligos, with 9330‐Gy14 chr5 containing only 1280 and 1152 oligos, respectively (Table S1).

FISH signal quality depends on the number of fluorophores that can be captured. Increasing the number of fluorophores carried by a single oligo can compensate for the insufficient oligo density. Based on this principle, we reconstructed each oligo by ligating two 35‐bp non‐genomic SE on both flanks of the oligo. The SE can recruit fluorophore‐labelled secondary oligos during FISH hybridization to increase the number of fluorophores carried by a single oligo. Specifically, the oligo flanks of hap9930‐hapGy14 and hap9930‐hapCuc64 are attached to SE1/2, SE3/4, and primer‐F/R sequences, respectively, to distinguish individual chromosomes (Figure 1a; Table S1). The reverse‐stranded oligos of 9930‐chr5 were generated to compare the differences in secondary oligos recruitment between double‐stranded (ds) oligos and single‐stranded oligos. Four small fragment sequences (4, 8, 12, and 25 kb) on 9930‐chr7 span 80, 150, 250, and 520 oligos to test the potential of our proposed method in localizing small fragments. A total of 106 810 designed oligos were synthesized into an oligo‐library (Genscript Biotech, Nanjing, China).

### Synthesis of ss/ds‐oligo probes and secondary oligos

Here, we describe two oligo synthesis strategies, ss‐ and ds‐oligo probes. The oligo‐library was divided into Multiple chromosomes or fragments‐specific sub‐libraries by PCR amplification using unlabelled P‐F/R primers. The F/R‐ss‐oligo probes were synthesized as follows. The forward‐ and reverse‐strand of chr5 sub‐libraries were used as templates for PCR amplification by 5′‐TAMRA‐labelled 20 bp SE1 and 5′‐phosphorylation‐modified 20 bp SE2 primers, respectively. Two units of lambda exonuclease (M0262; New England Biolabs, Ipswich, MA, USA) were added to the two 50 μL PCR reactions obtained, respectively. These reactions were incubated at 37 °C for 20 min in a programmable thermocycler and then stopped at 75 °C for 10 min. The digestion products were concentrated by GeneJET PCR Purification kit and eluted with 20 μL solution. The final solutions after quantification by spectrophotometry are the ss‐oligo probes for the forward‐ and reverse‐strand of 9930‐chr5. The ds‐oligo probes were synthesized as follows. The sub‐libraries were used as templates for PCR amplification by TAMRA‐ or FAM‐labelled 20 bp SE primers. The PCR products were purified with the GeneJET PCR Purification kit to obtain chromosome‐specific ds‐oligo probes. All PCR and concentration procedures were performed as previously described (Zhao *et al*., 2021).

For secondary oligos, 2 bp on either side of the near‐genomic sequence was deleted to release the space occupied by the fluorophores. The remaining 33bp_SE sequences and their complementary sequences were modified with TAMRA or FAM at the 5′ and 3′ ends, respectively (Tsingke Biotech, Nanjing, China). Eight secondary oligo libraries were finally obtained and premixed into a 10 μM working solution.

### Oligo‐painting and image processing

The oligo‐painting protocol was performed as described previously with some modifications (Zhao *et al*., 2021, 2022). Specifically, the hybridization mixtures contained 10 μL of 100% Formamide deionized, 2 μL of 2 × SSC, 1 μL of ss‐oligo or ds‐oligo probes, 0 μL of secondary oligos or 1 μL of 33 bp secondary oligos mix (two or four or eight), and 4 μL of 50% dextran sulphate. Denatured (ds‐oligo probes) or non‐denatured (ss‐oligo probes) mixtures were covered onto prepared slides and then placed at 37 °C for overnight treatment. The subsequent slides washing and FISH capture were performed as described previously (Zhao *et al*., 2022).

The FISH images were separated and merged using Adobe Photoshop software. Fluorescence intensity was quantified by ImageJ software. We measured the distance of each chromosome exchange point to the end of the corresponding chromosome arm and the length of the corresponding arm using ImageJ. Each CO was assigned to the corresponding chromosome interval by dividing the measured distance by the total length of the chromosome arm.

### Sequencing and genotyping

Genomic DNA was extracted from two hardwickii‐9930F_2_ plants. We generated an average of 10 × coverage of 150 bp pair‐end sequences. Clean reads were mapped to the 9930v3 genome by BWA MEM with default parameters (Li and Durbin, 2009). Reads with mapping quality ≥30 were retained by SAMtools. SNPs were detected by the freebayes as previously described (do Vale Martins *et al*., 2019). Genotypes of each SNP locus were extracted from the filtered vcf file. The numbers of SNPs were counted in 10 kb windows. Genotypes and percentage of SNPs within each window were analysed qualitatively. The windows were defined as homozygous hardwickii genotype when SNP are 1/1 and more than 90% in the window. The windows were defined homozygous hardwickii genotype when SNP are 1/1 and more than 90% in the window, homozygous 9930 genotype when SNP was close to zero in the window and heterozygous genotype when SNP is 0/1. We extracted the sequence variants of gy14 and hardwickii relative to 9930 (SNPs and InDels) as previously described (Li *et al*., 2022).

## Conflict of interest

The authors declare no competing interests.

## Author contributions

J.C., Q.L., and Q.Z. conceived the research. Q.Z. designed and performed the experiments. Z.X. and Z.L. participated in the synthesis of oligo probes. C.C., Y.W., and X.F. contributed to Bioinformatic support. Q.Z., Q.L., and J.C. wrote the manuscript. All authors read and approved the final article.

## Supporting information


**Figure S1** Development of enhanced haplotype oligo‐painting libraries and two strategies for probe synthesis.
**Figure S2** Positioning of four small chr7 fragments based on enhanced oligo‐painting.
**Figure S3** Differential painting of homologous chromosomes of Gy14‐9930F_1_ hybrids (G9F_1_).
**Figure S4** Differential painting of homologous chromosomes of 9930‐hardwickii F_1_ hybrids (9hF_1_).
**Figure S5** Recombination landscapes of seven homologous chromosomes based on EHOP in Gy14‐9930F_2_ population.
**Figure S6** Chromosome evolution diagram of cultivated 9930 and ancestral hardwickii.
**Figure S7** Recombination landscapes of seven homologous chromosomes based on EHOP in hardwickii‐9930F_2_ population.
**Figure S8** Validation of chr3 recombination events based on sequencing analysis in two selected hardwickii‐9930F_2_ plants.

**Table S1** Information regarding cucumber oligo‐library in this study.
**Table S2** Arm ratios of cucumber chromosomes and COs frequency on arms.

## Data Availability

List of oligos, signal enhancers, and primers used in this study can be found in Table S1.
