# Supplementary material for: Meiotic crossovers revealed by differential visualization of homologous chromosomes using enhanced haplotype oligo‐painting in cucumber
Source: Plant Biotechnol J. 2024 Dec 11;23(3):887–99. doi: 10.1111/pbi.14546 (PMC11869176; doi:10.1111/pbi.14546)
Supplement: Supplementary file 1 — Figure S1 Development of enhanced haplotype oligo‐painting libraries and two strategies for probe synthesis. Figure S2 Positioning of four small chr7 fragments based on enhanced oligo‐painting. Figure S3 Differential painting of homologous chromosomes of Gy14‐9930F1 hybrids (G9F1). Figure S4 Differential painting of homologous chromosomes of 9930‐hardwickii F1 hybrids (9hF1). Figure S5 Recombination landscapes of seven homologous chromosomes based on EHOP in Gy14‐9930F2 population. Figure S6 Chromosome evolution diagram of cultivated 9930 and ancestral hardwickii. Figure S7 Recombination landscapes of seven homologous chromosomes based on EHOP in hardwickii‐9930F2 population. Figure S8 Validation of chr3 recombination events based on sequencing analysis in two selected hardwickii‐9930F2 plants. [file PBI-23-887-s002.docx]

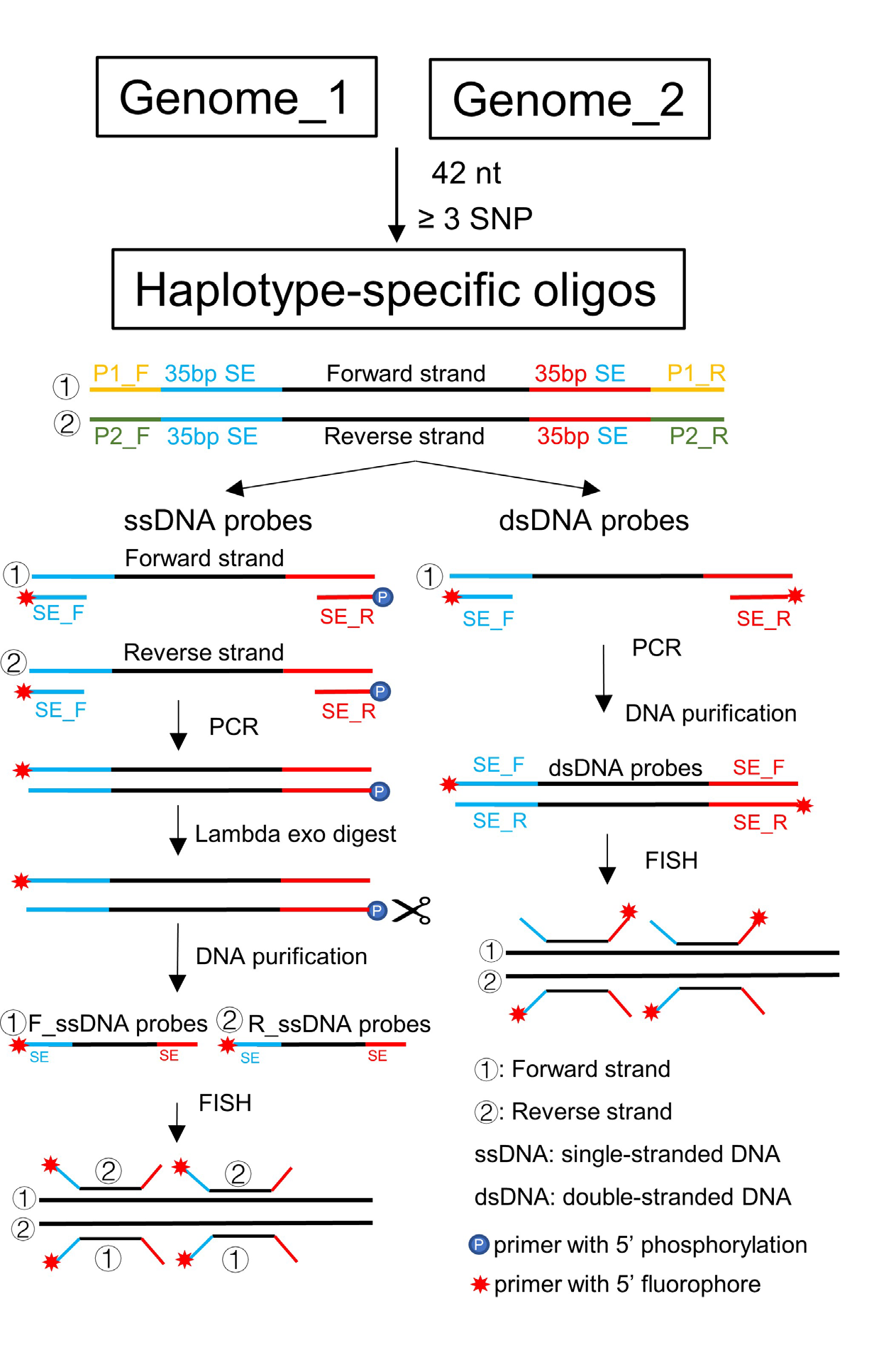
**Supporting Information**

**Figure S1** Development of enhanced haplotype oligo-painting libraries and two strategies for probe synthesis.

Haplotype-specific oligos were reconstructed after screening by the software process. Each oligo was flanked by 35bp non-genomic enhancement sequences (SE) and PCR amplification primers (P_R/F). Each chromosome-specific sub-library was amplified from the total oligos library by using different P_R/F primers for PCR. Double-stranded oligo probes were obtained by amplification using fluorescently-labelled 20bp SE primers based on the sub-library as a template. To obtain a single-stranded probe, the forward and reverse strands of chr5 were amplified separately by PCR amplification with fluorescence- and phosphorylation-modified SE primers. The phosphorylated strands were digested by Lambda exo and subsequent DNA purification to obtain single-stranded oligo probes for both the forward and reverse strands.


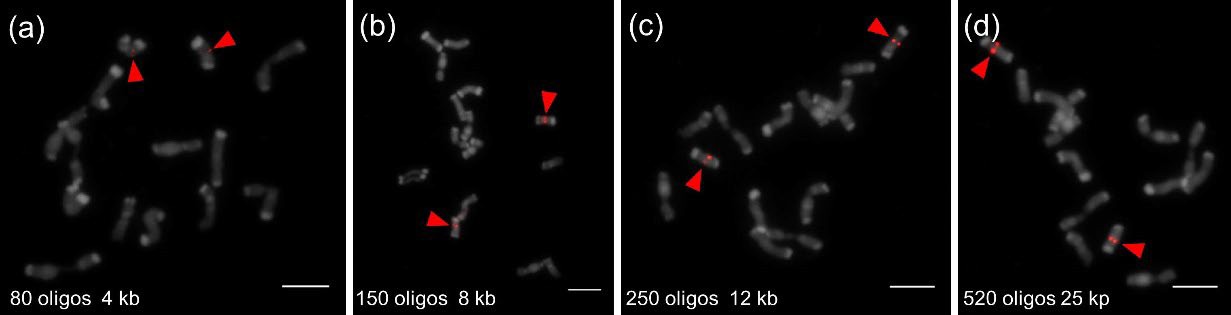


**Figure S2** Positioning of four small chr7 fragments based on enhanced oligo-painting.


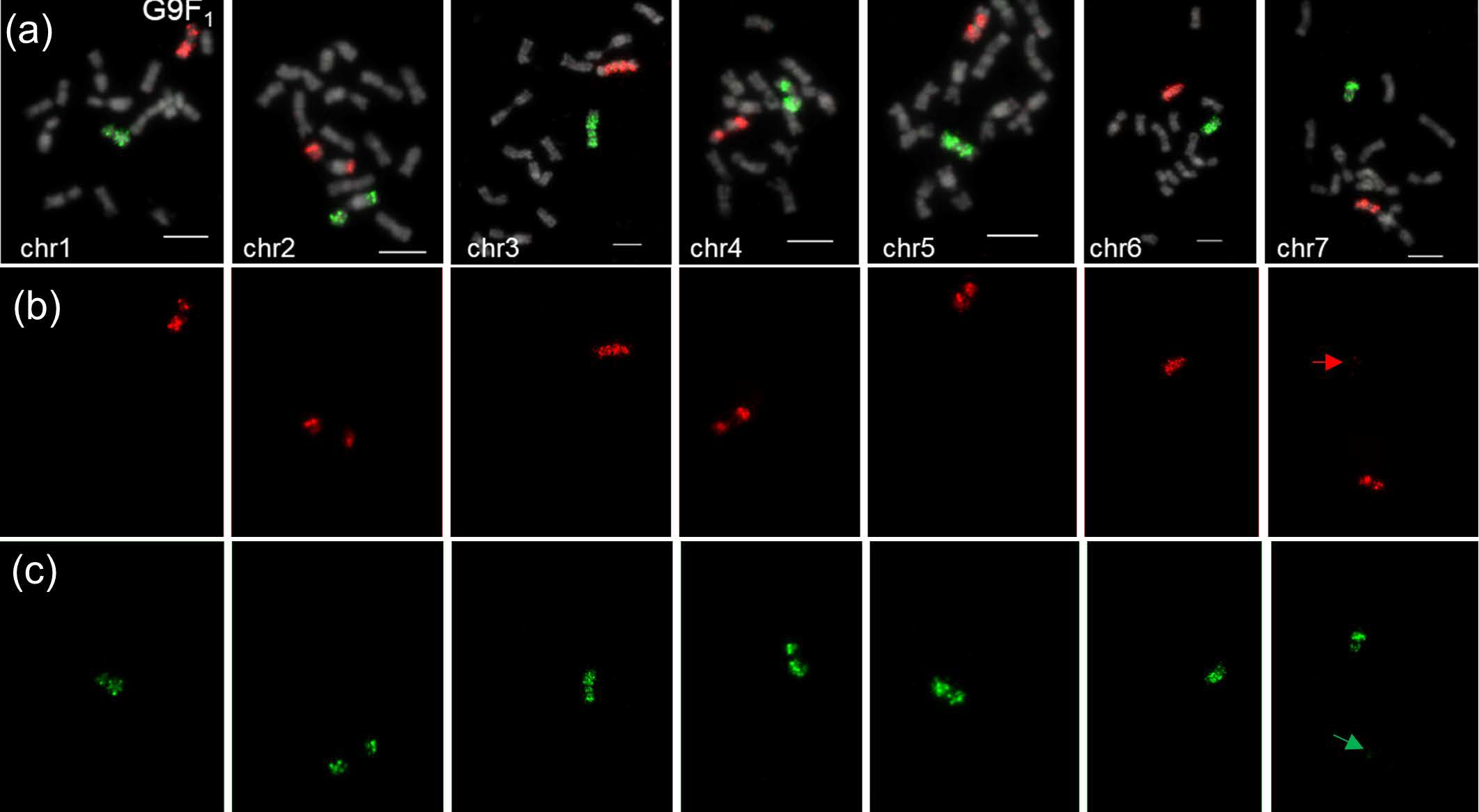


# Figure S3 Differential painting of homologous chromosomes of Gy14-9930 F1 hybrids (G9F1).

(a) Differential painting of seven homologous chromosomes by enhanced haplotype oligo-painting (EHOP) on metaphase chromosomes. Oligo-FISH probes specific to the 9930 haplotype were detected in red color. Oligo-FISH probes specific to the Gy14 haplotype were detected in green color. (b) Digitally separated red FISH signals derived from the 9930-specific probes. (c) Digitally separated green FISH signals derived from the Gy14-specific probes. Red and green arrows indicate cross hybridization signals. Bars=5 μm.


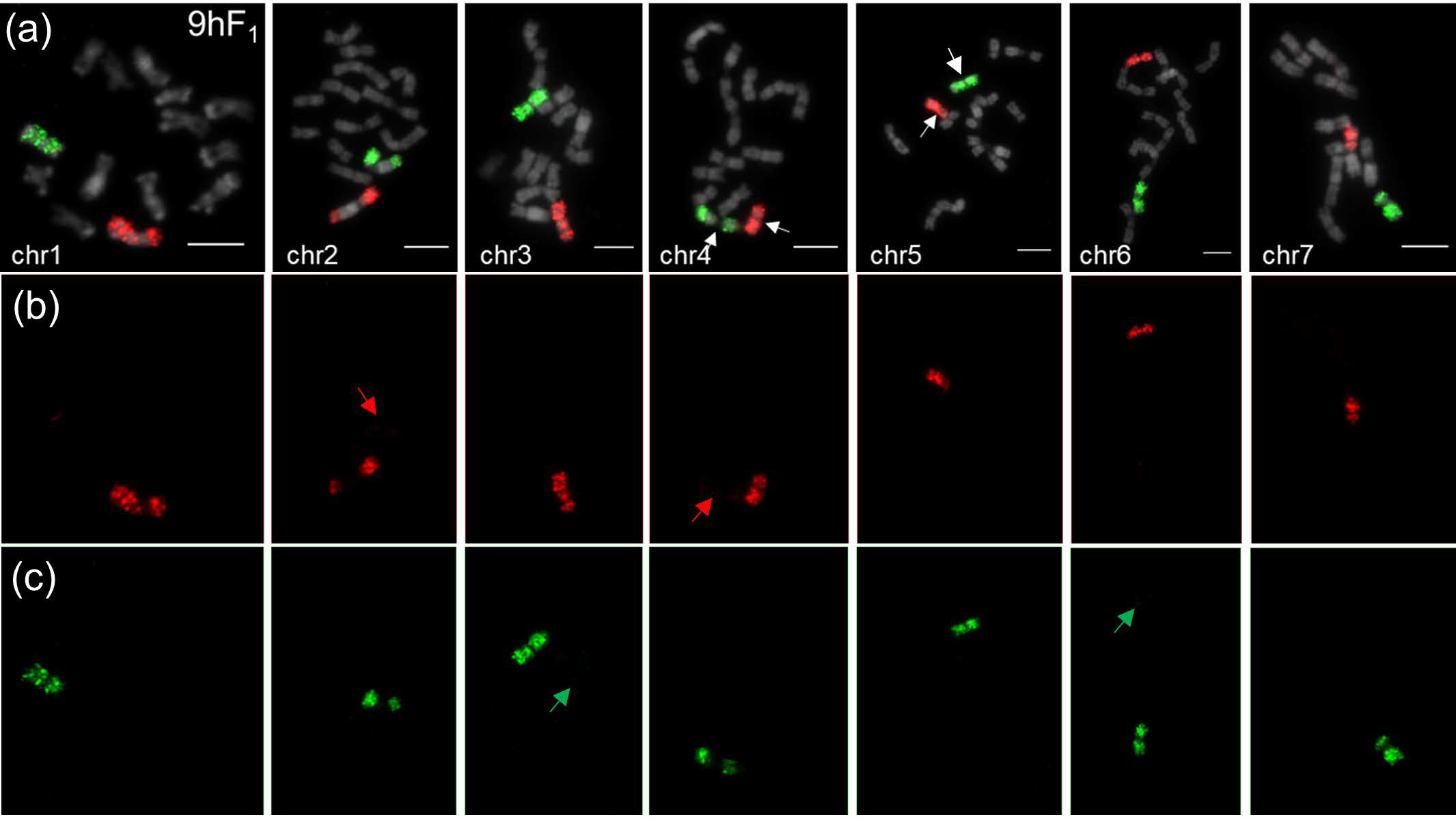


# Figure S4 Differential painting of homologous chromosomes of 9930-hardwickii F1 hybrids (9hF1).

(a) Differential painting of seven homologous chromosomes by enhanced haplotype oligo-painting (EHOP) on metaphase chromosomes. Oligo-FISH probes specific to the hardwickii haplotype were detected in red color. Oligo-FISH probes specific to the 9930 haplotype were detected in green color. (b) Digitally separated red FISH signals derived from the hardwickii-specific probes. (c) Digitally separated green FISH signals derived from the 9930-specific probes. White arrows indicate karyotypic differences in chr4 and chr5 due to domestication inversions. Red and green arrows indicate cross hybridization signals. Bars=5 μm.


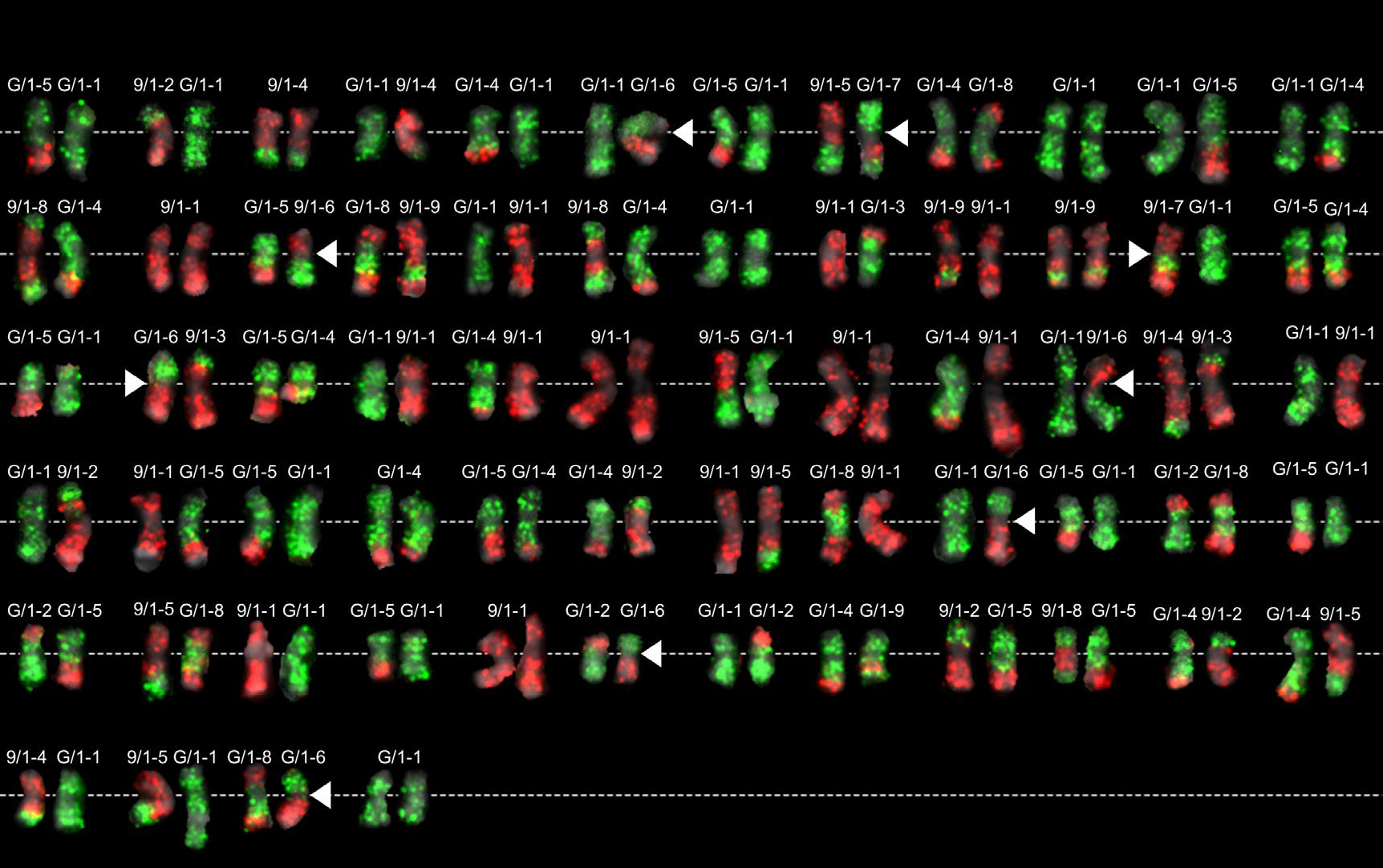

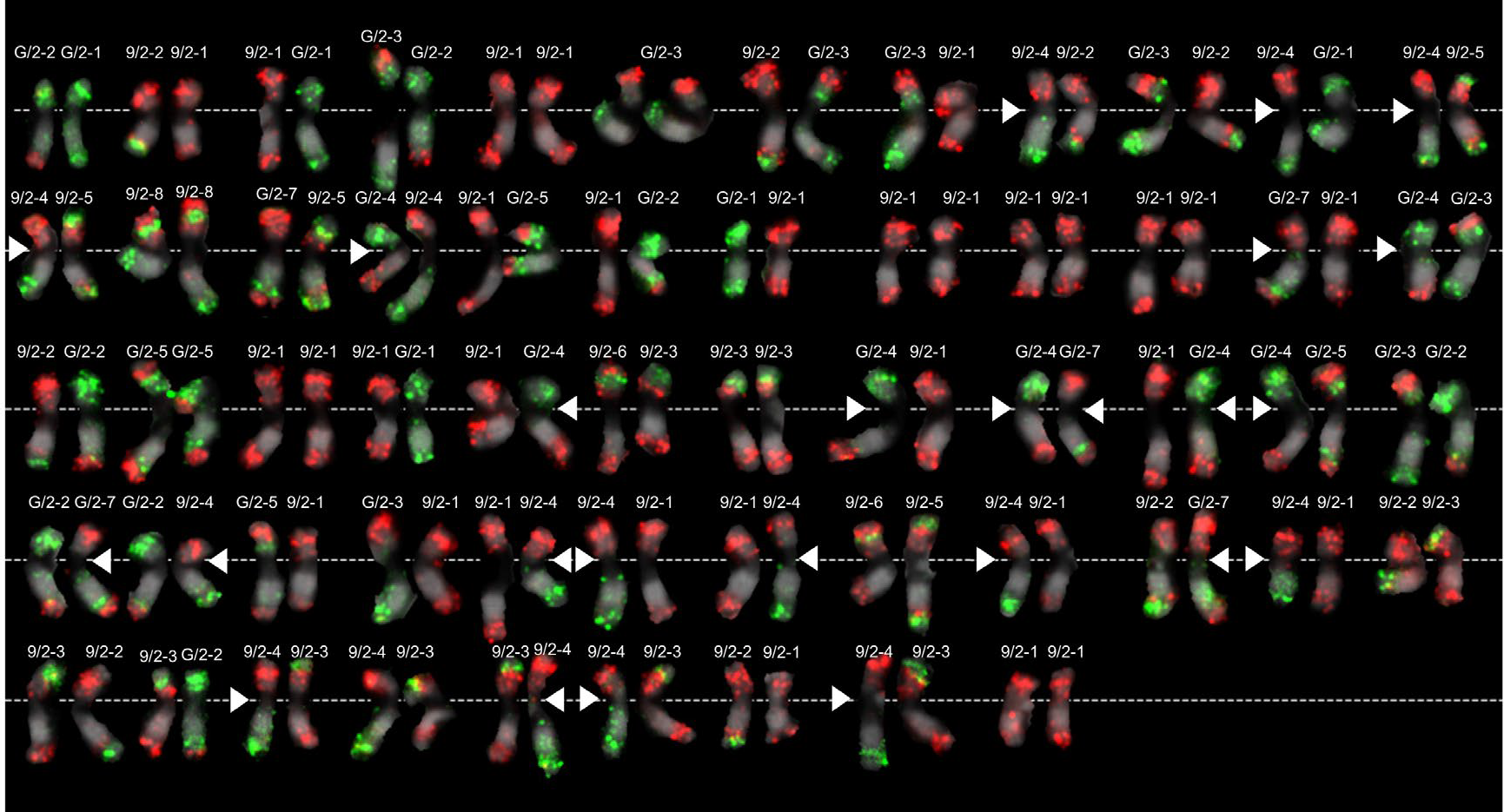

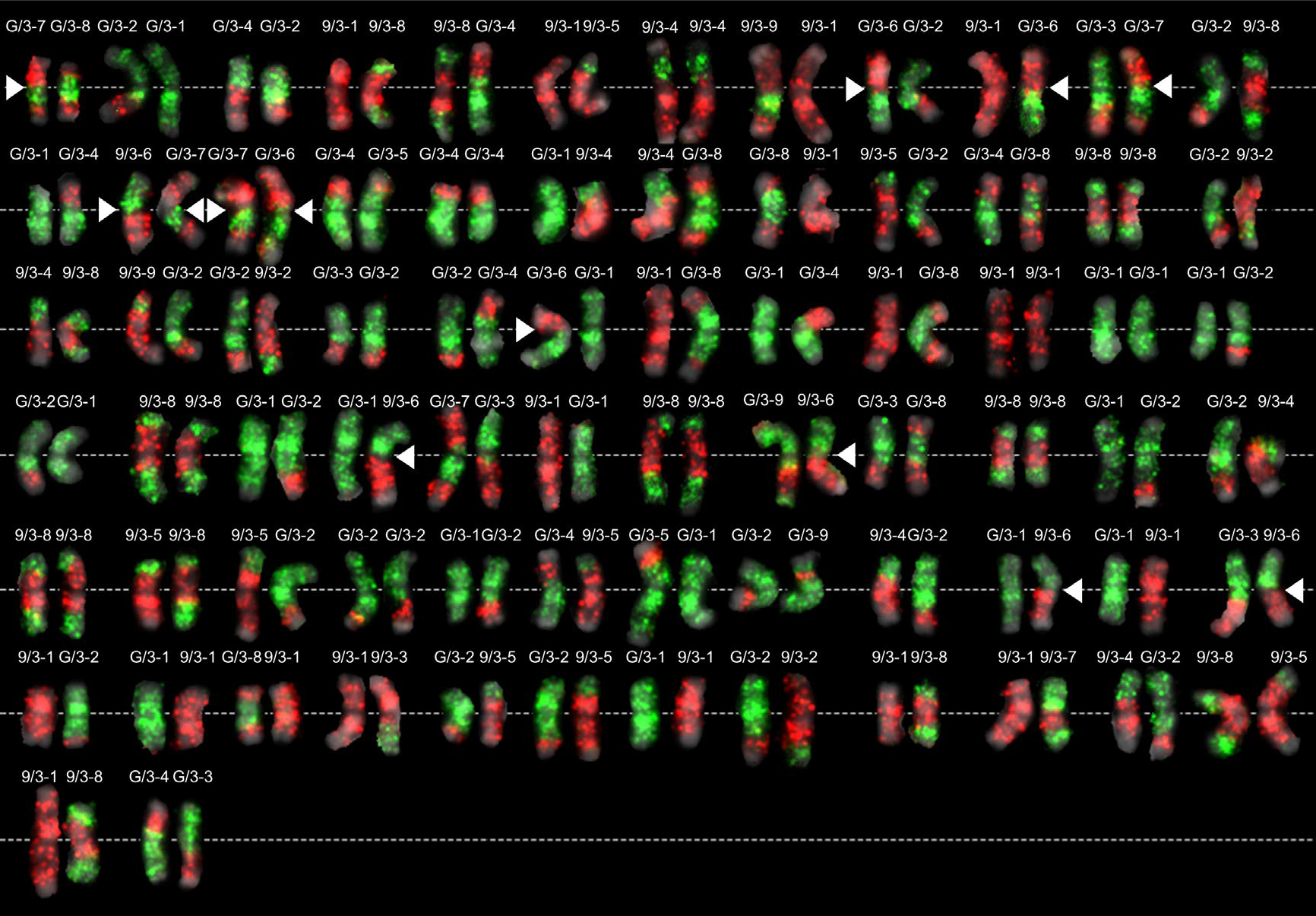

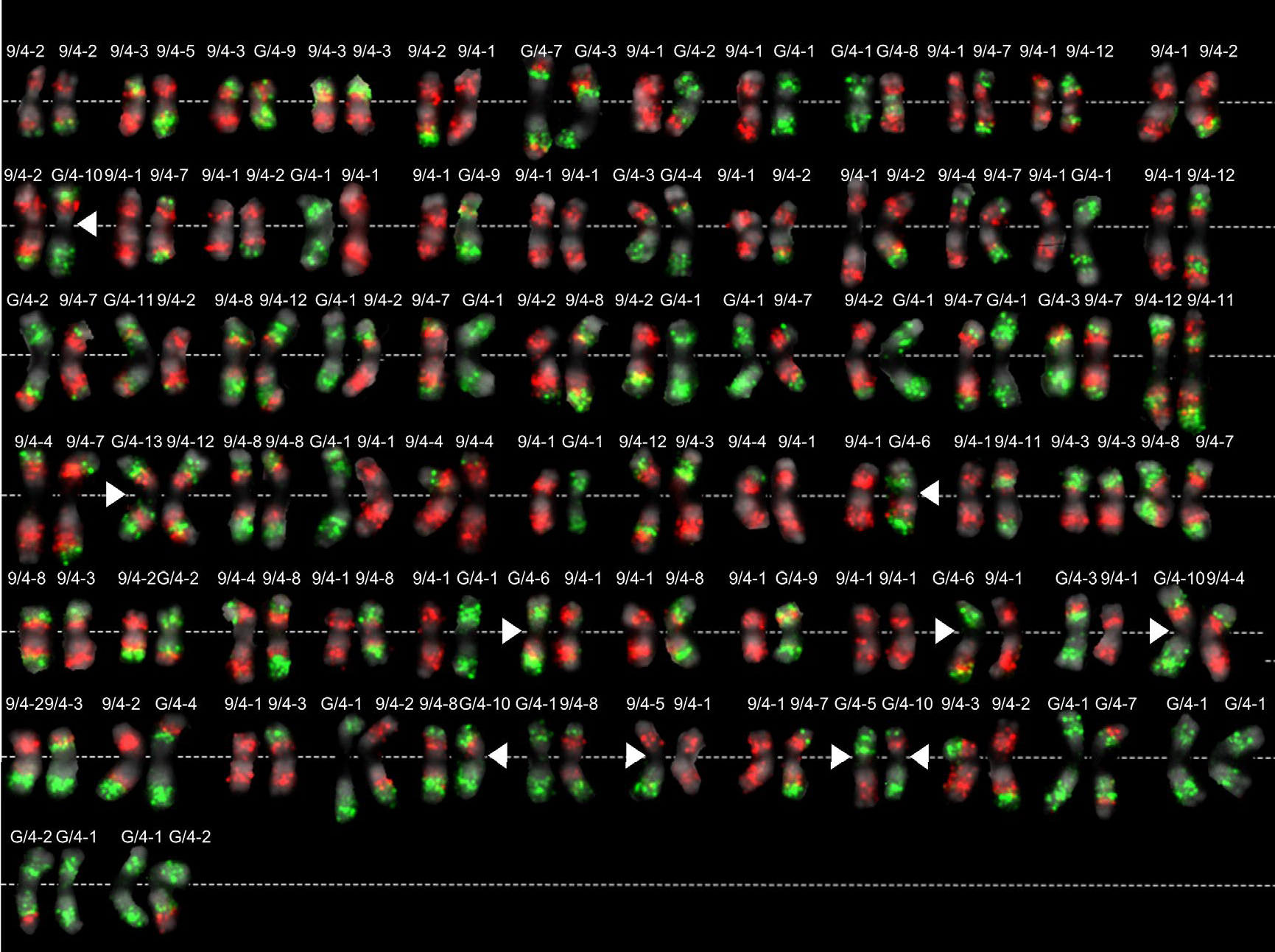

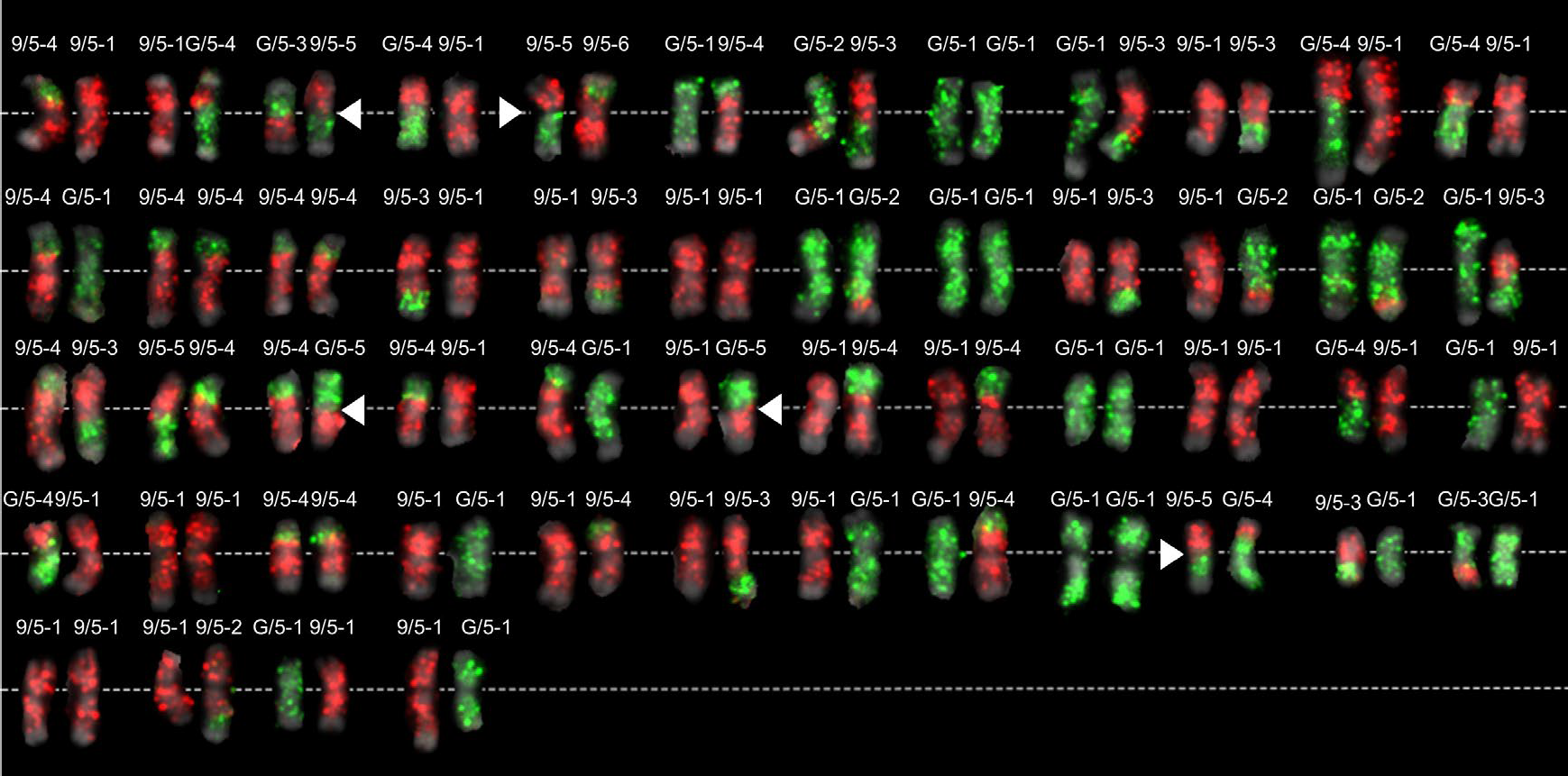

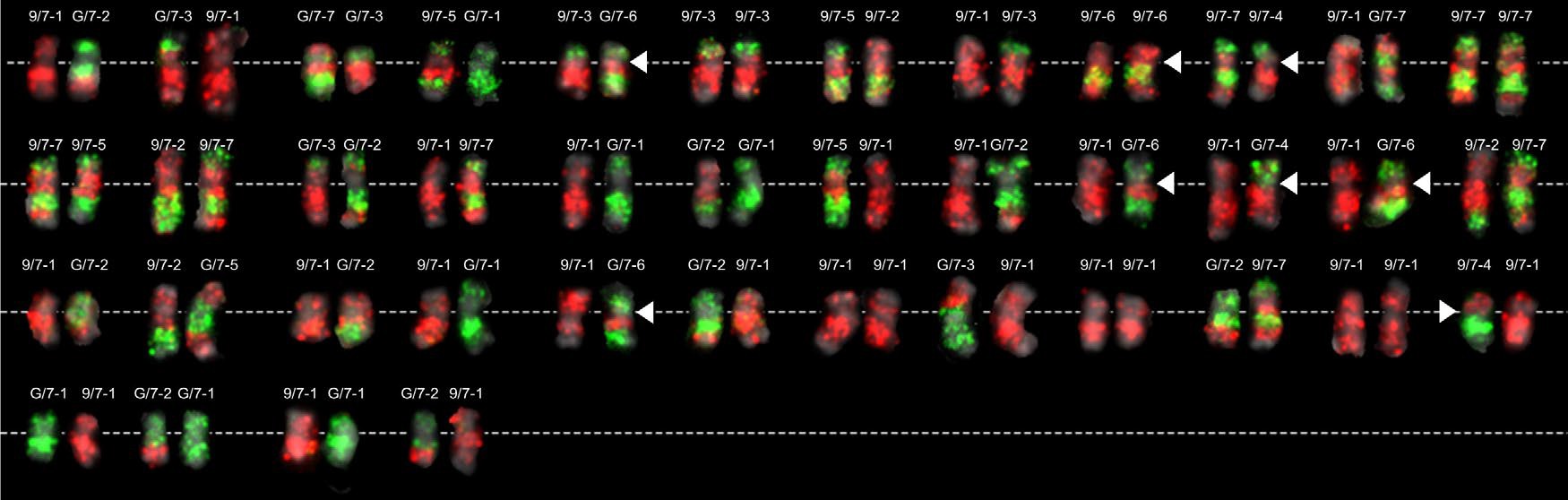

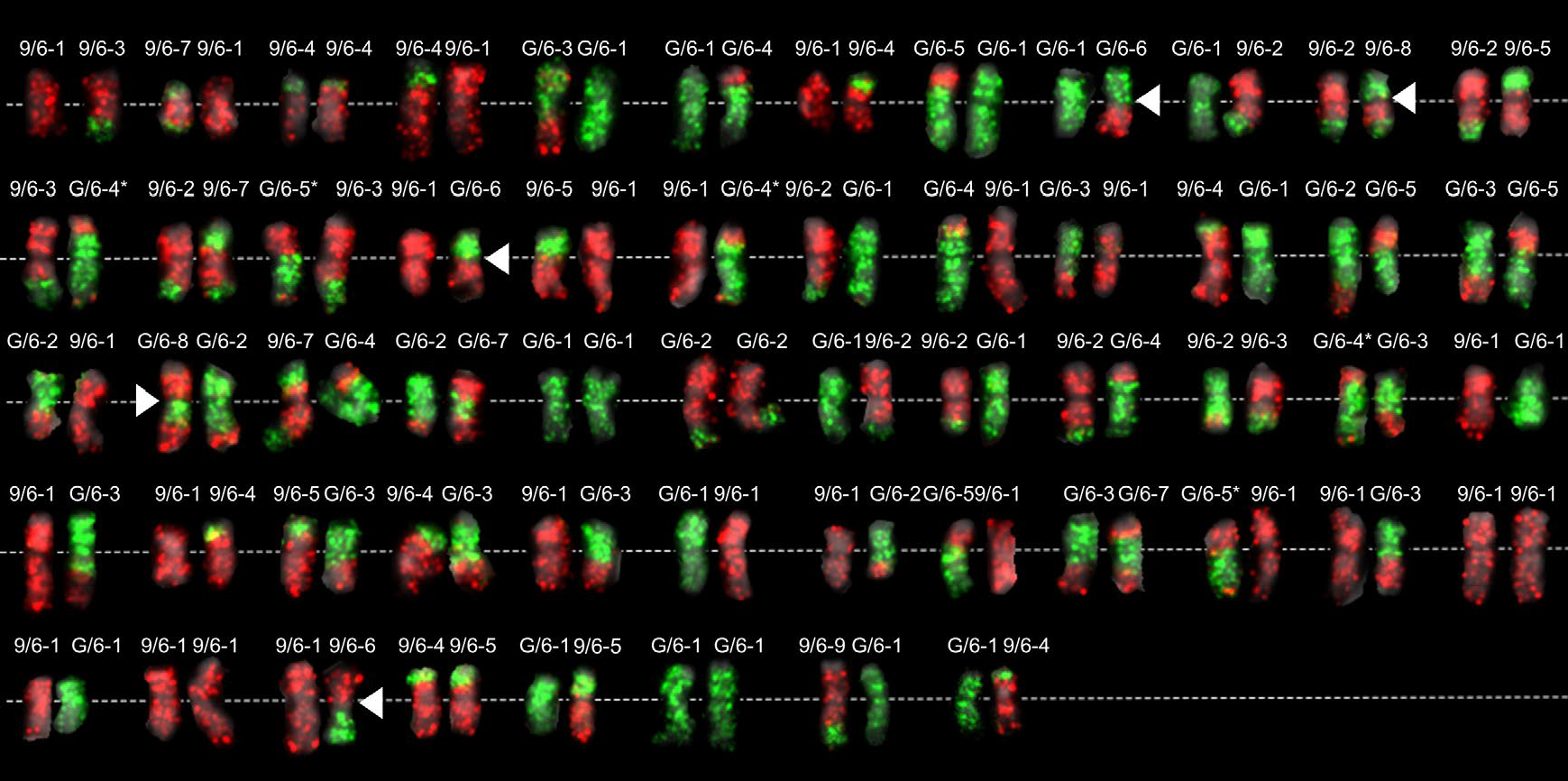


**Figure S5** Recombination landscapes of seven homologous chromosomes based on EHOP in Gy14-9930 F2 population.

White dashed lines mark the positions of the cytological centromeres. White arrows indicate that the observed chromosome exchange positions are in cytological centromere (primary constriction) regions. 9930-chr1 was abbreviated to 9/1, and the other chromosomes follow suit. Gy-chr1 was abbreviated to G/1, and the other chromosomes follow suit. Grouping similar recombinant chromosomes into one pattern.


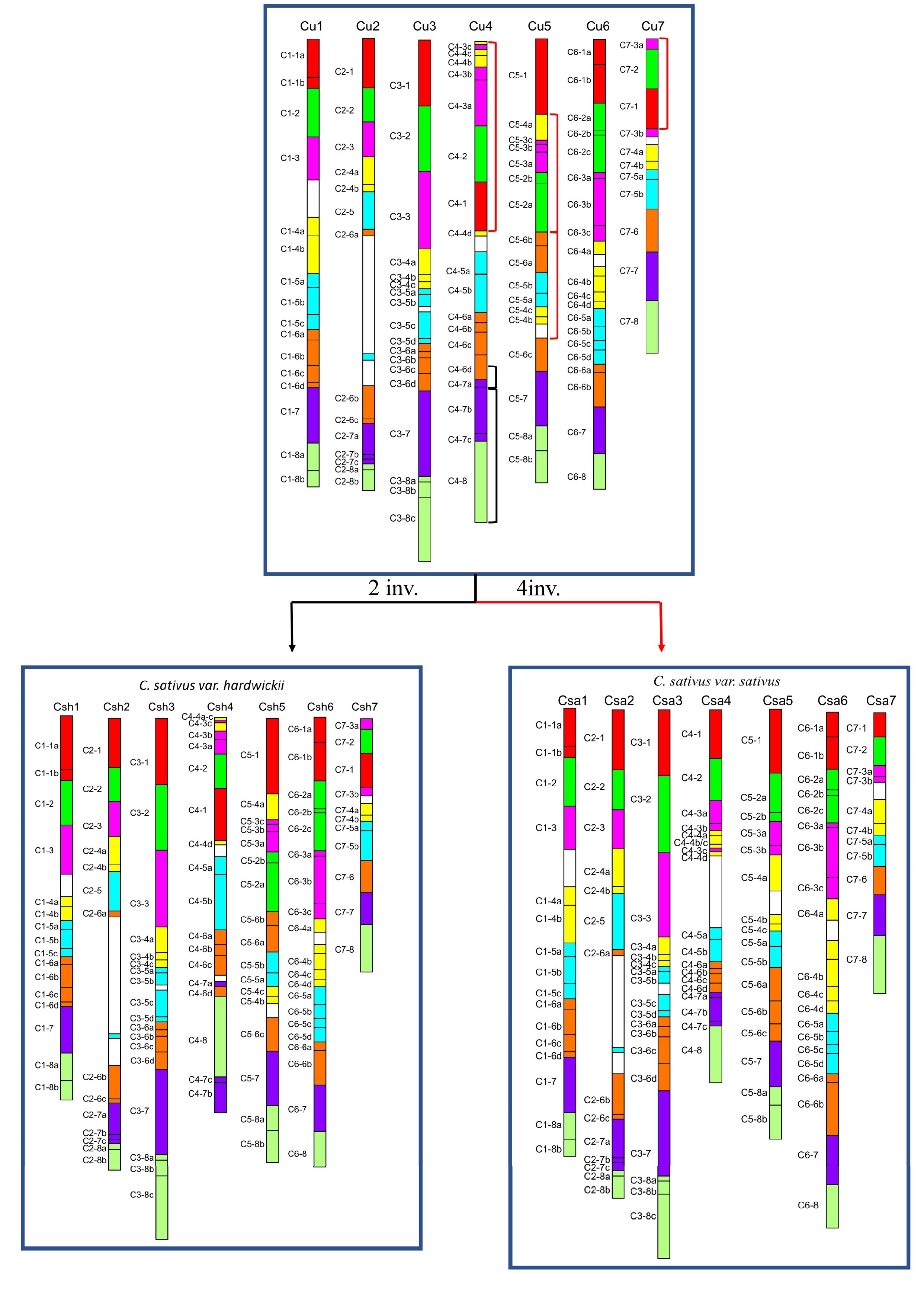


**Figure S6** Chromosome evolution diagram of cultivated 9930 and ancestral hardwickii. This diagram was quoted from our previously published article “Reconstruction of ancestral karyotype illuminates chromosome evolution in the genus Cucumis” and mainly used to illustrate the existence of inversion events on chr4, chr5, and chr7.


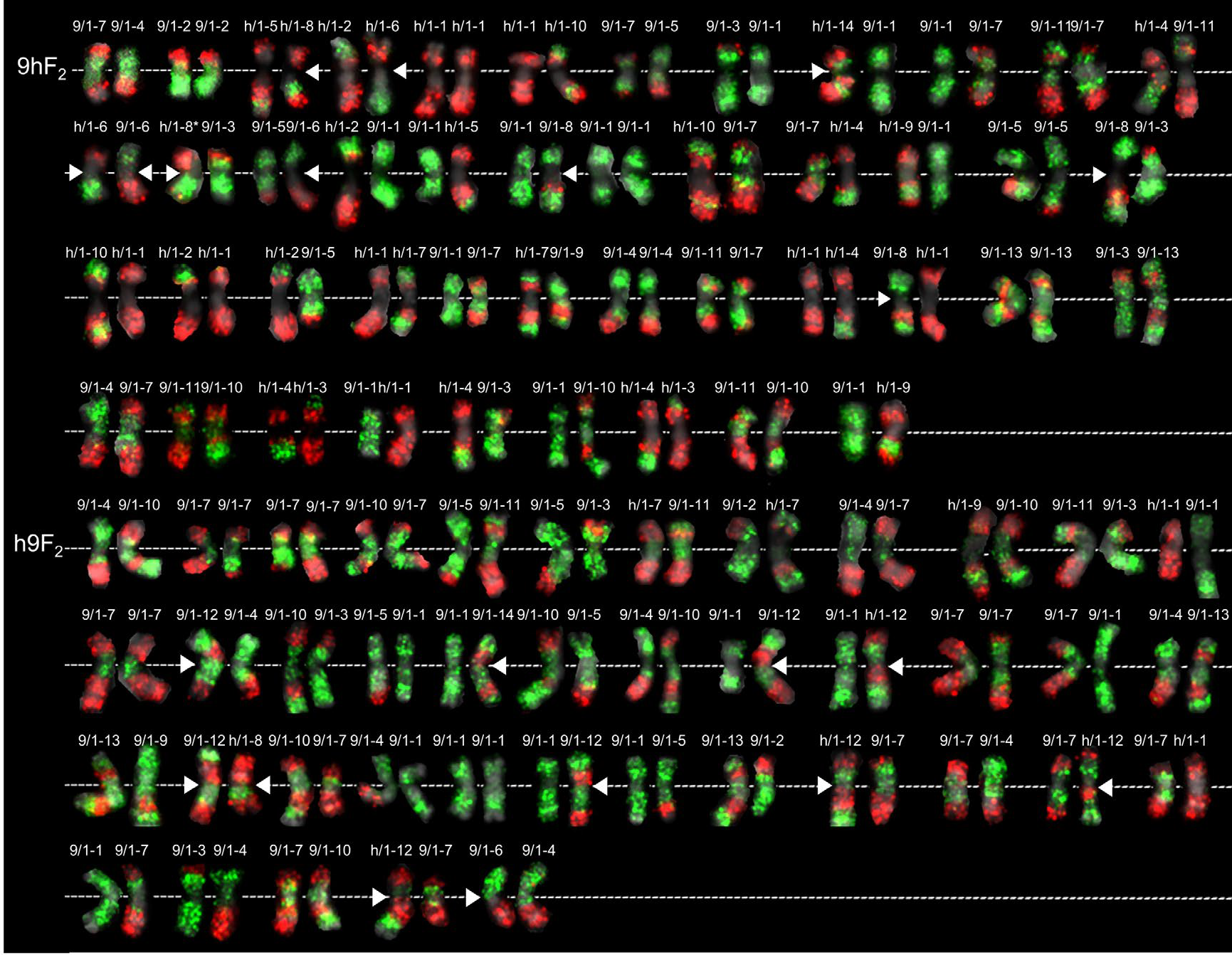


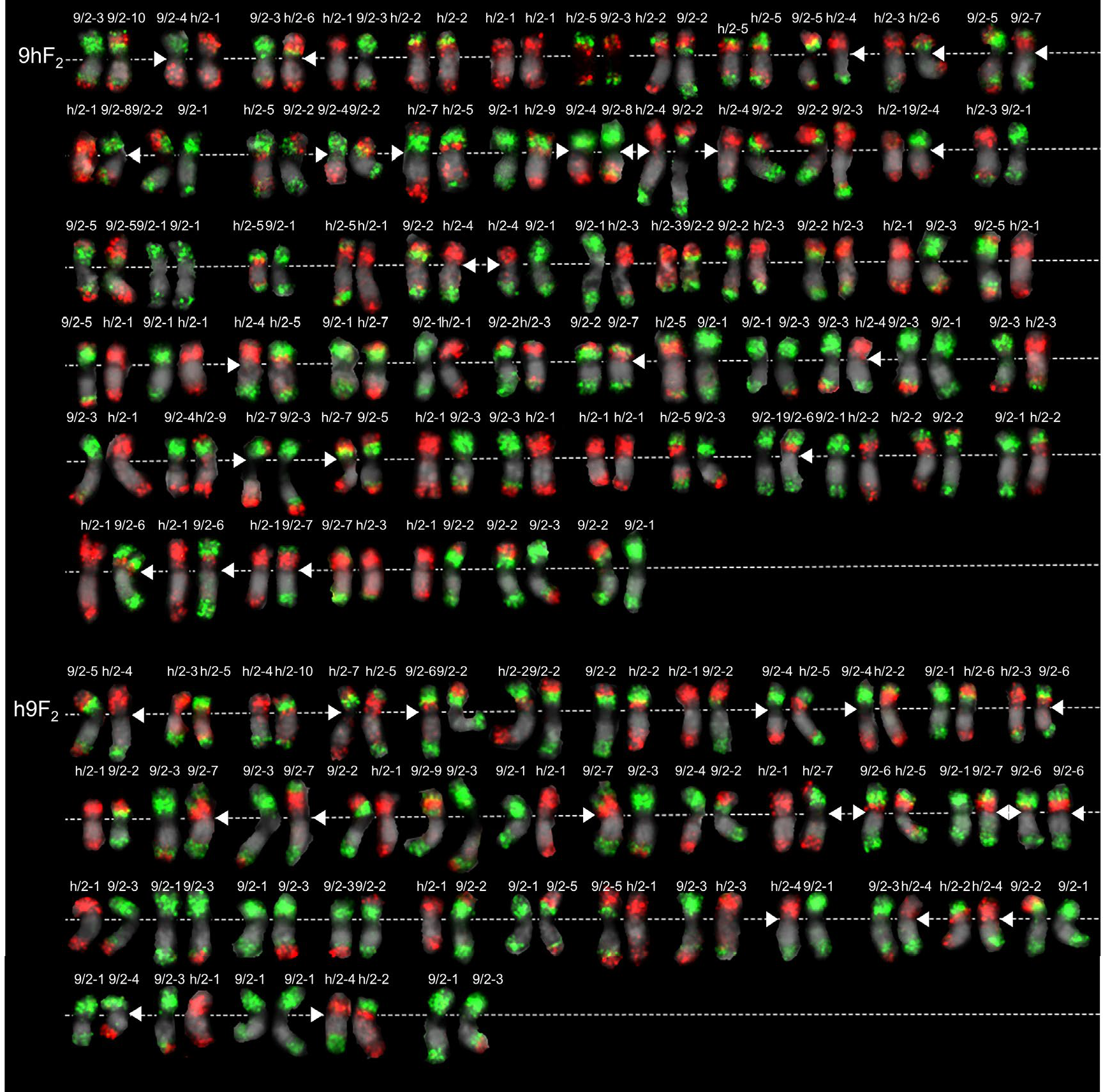


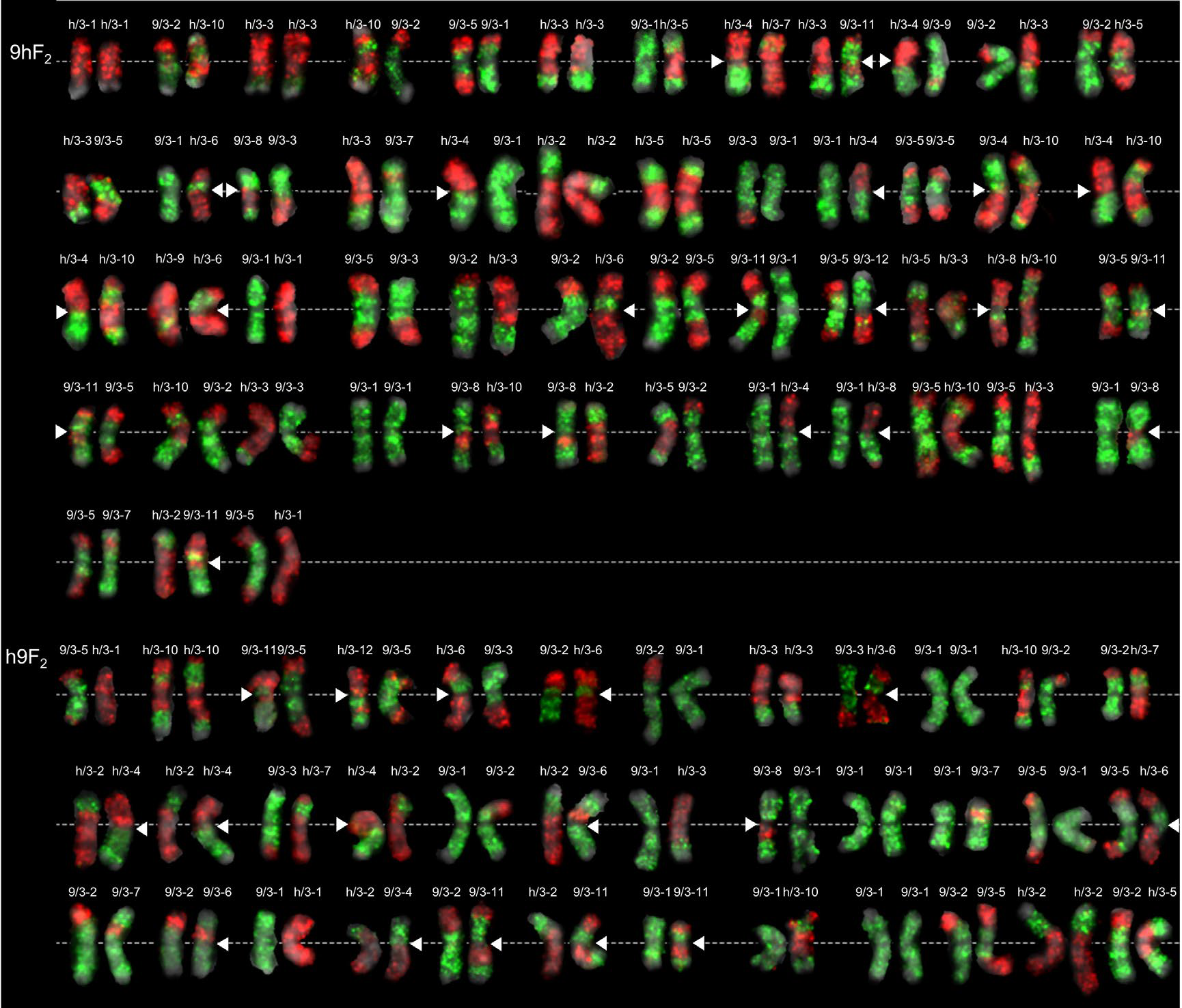


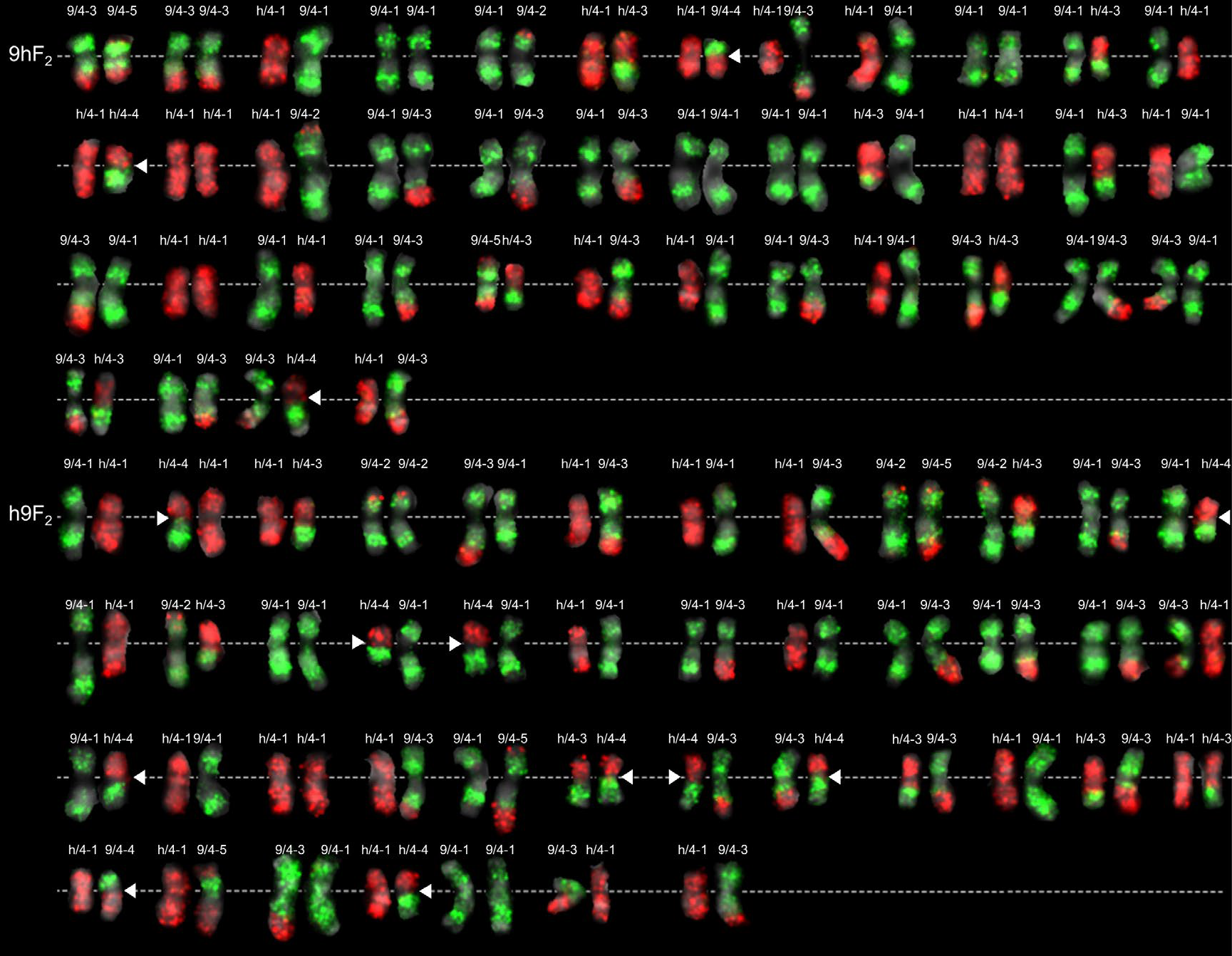


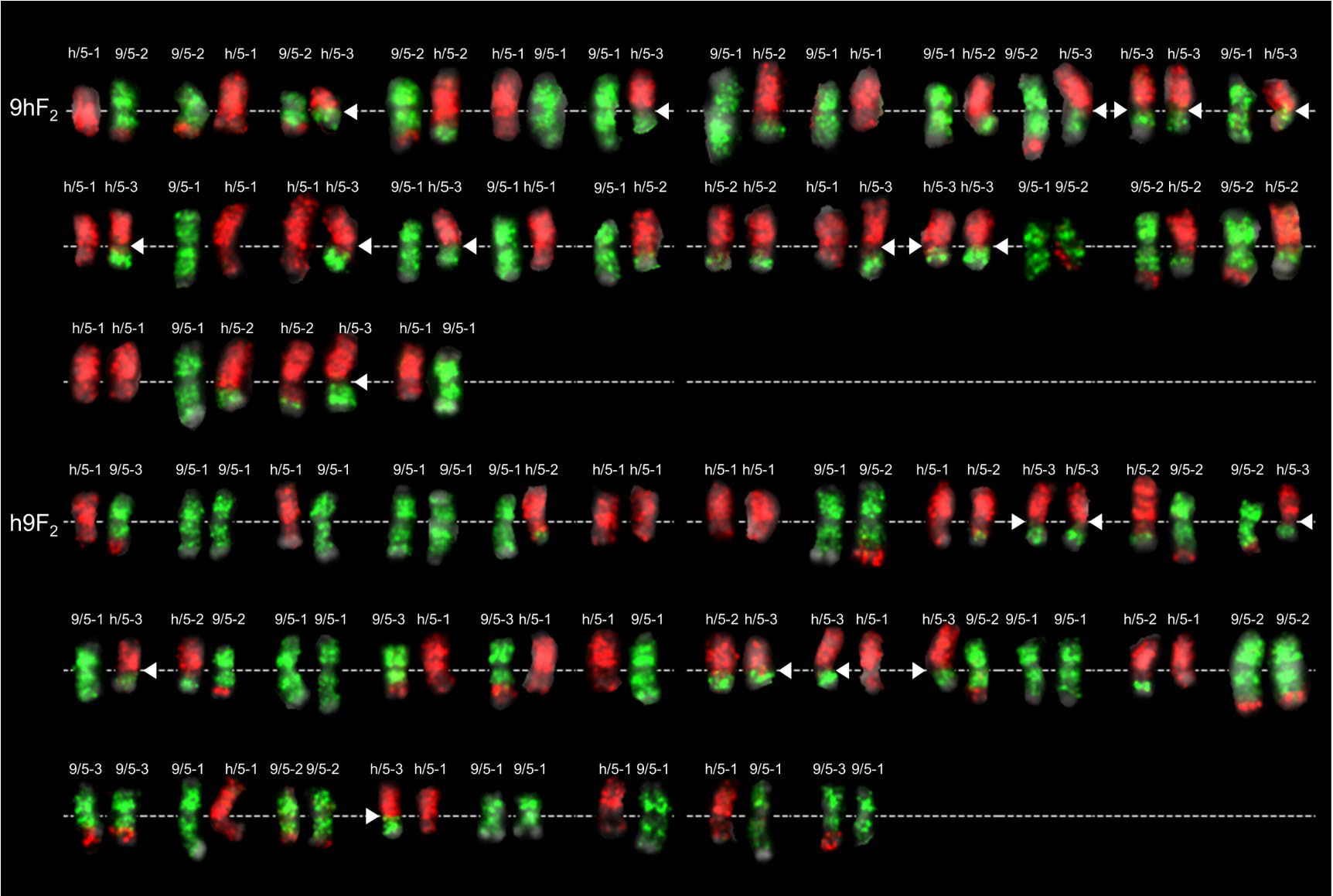

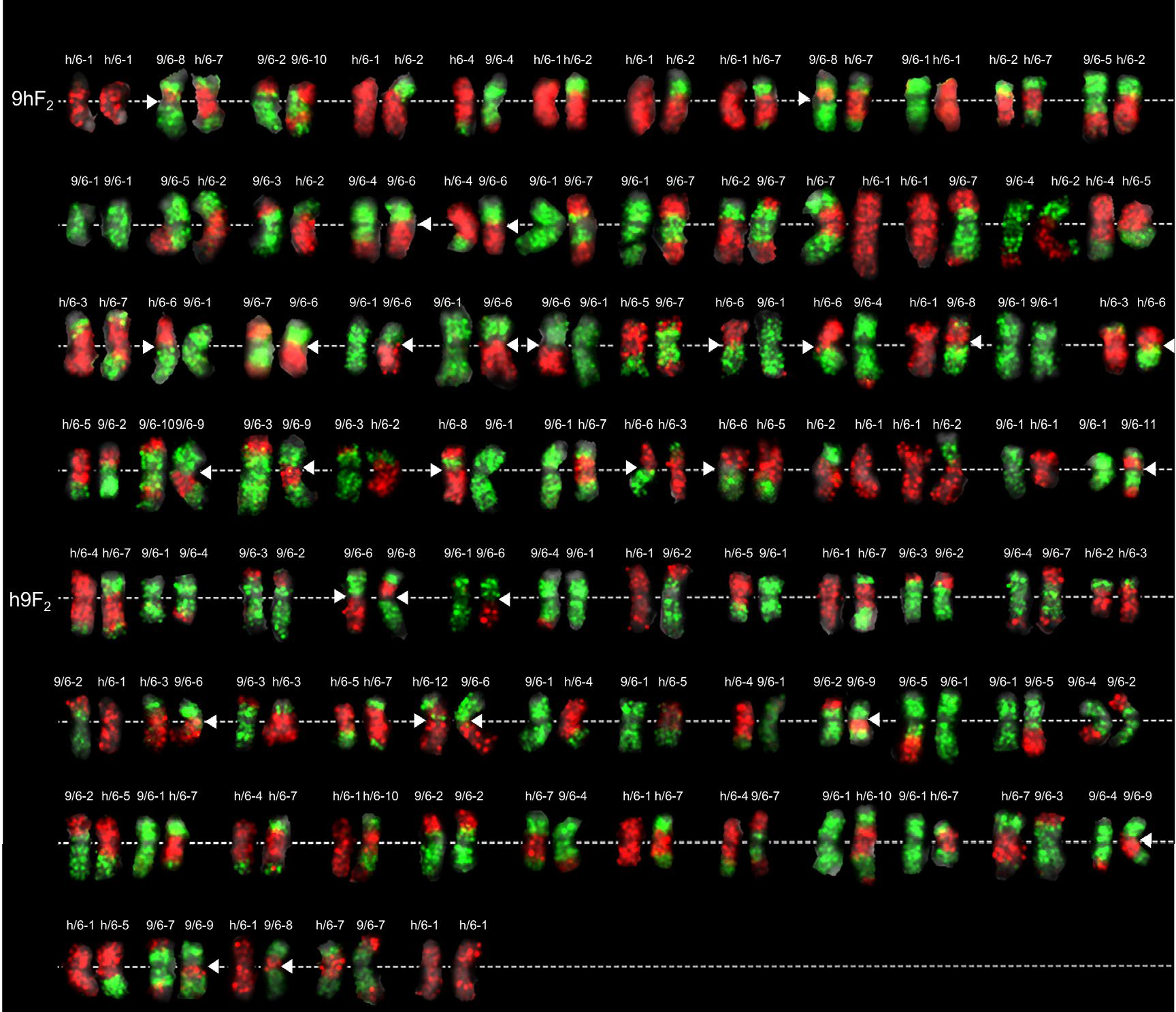


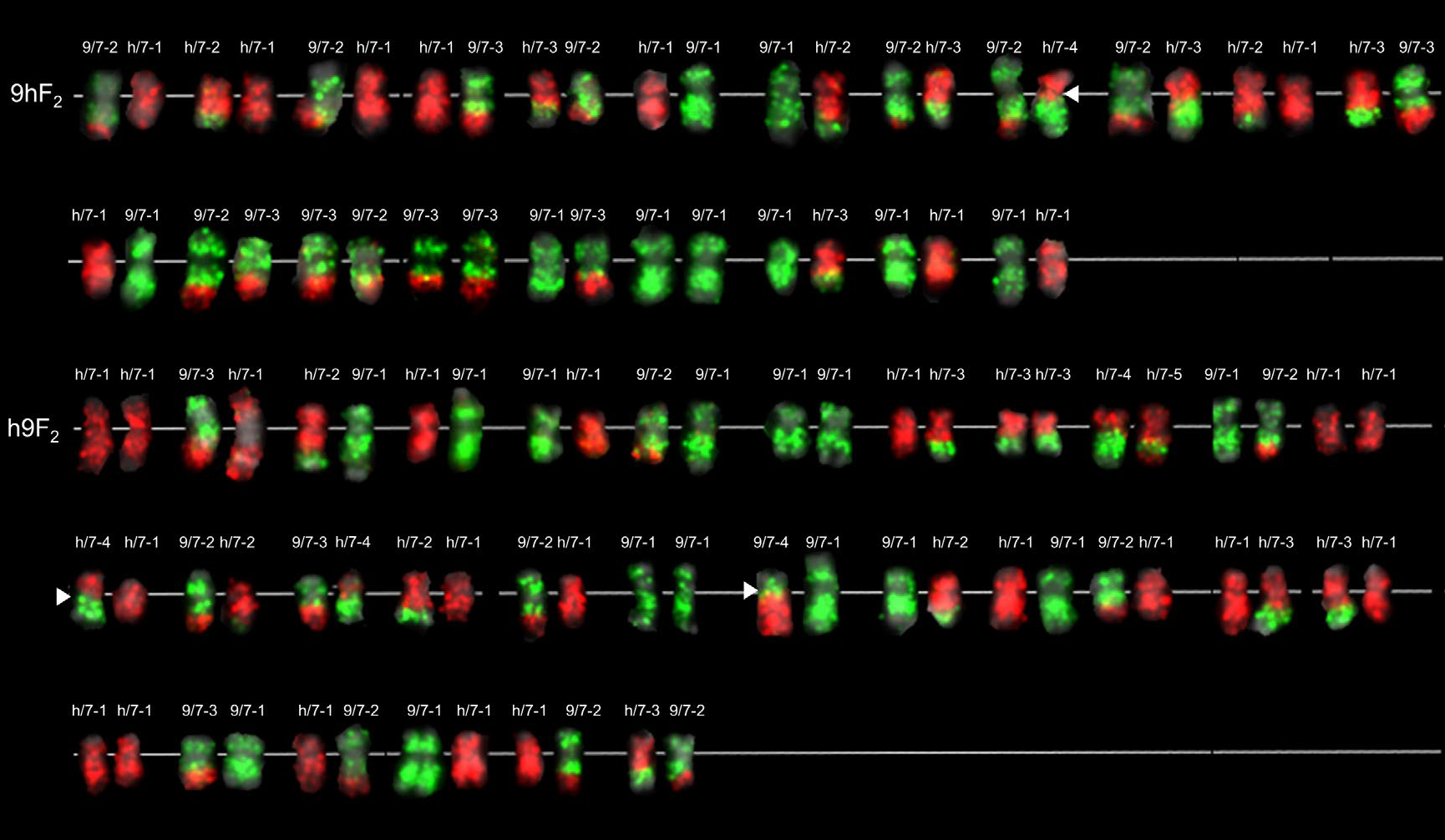


**Figure S7** Recombination landscapes of seven homologous chromosomes based on EHOP in hardwickii-9930 F2 population.

White dashed lines mark the positions of the cytological centromeres. White arrows indicate that the observed chromosome exchange positions are in cytological centromere (primary constriction) regions. 9930-chr1 was abbreviated to 9/1, and the other chromosomes follow suit. Hardwickii-chr1 was abbreviated to h/1, and the other chromosomes follow suit. Grouping similar recombinant chromosomes into one pattern.


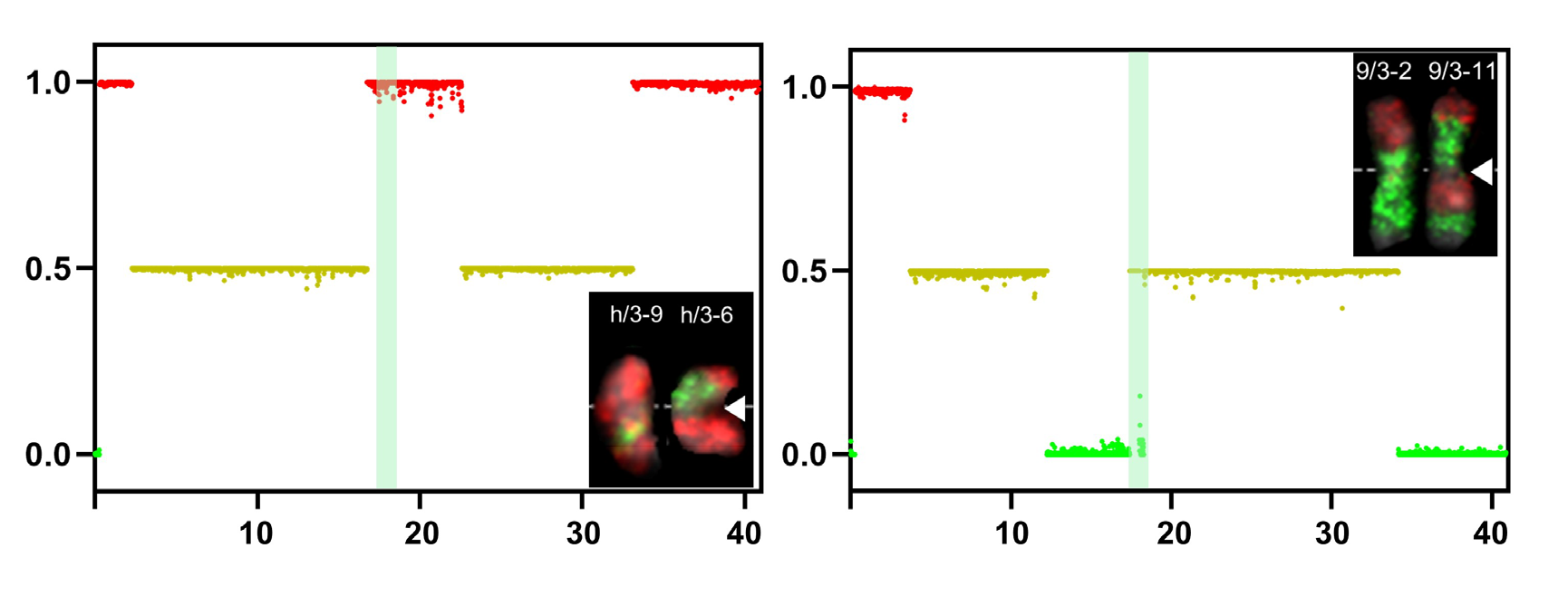


**Figure S8** Validation of chr3 recombination events based on sequencing analysis in two selected hardwickii-9930 F2 plants.

The *y*-axis of each sequencing profile represents the allele frequency of hardwickii (red) or 9930 (green). The *x*-axis represents the sequence position of chr3 (40.8 Mb). Yellow dots indicate the mix of red (hardwickii) and green (9930) dots. The light green boxes indicate the putative centromere regions.

| Chromosomes | Arm ratio | COs frequency on short arm | COs frequency on long arm |
| --- | --- | --- | --- |
| Chr1 | 1.30 ± 0.05 | 17.2% | 43.8% |
| Chr2 | 1.45 ± 0.06 | 34.2% | 27.2% |
| Chr3 | 1.09 ± 0.08 | 38.5% | 48.0% |
| Chr4 | 1.18 ± 0.04 | 49.3% | 45.3% |
| Chr5 | 1.12 ± 0.08 | 24.0% | 17.3% |
| Chr6 | 1. 34 ± 0.07 | 26.8% | 38.4% |
| Chr7 | 1.37 ± 0.05 | 28.8% | 53.8% |

**Table S2** Arm ratios of cucumber chromosomes and COs frequency on arms Arm ratio: length of the long arm/length of the short arm.
